# Beyond receptor activation: biased toll-like receptor signaling in periodontal inflammation and regeneration

**DOI:** 10.3389/fimmu.2026.1852272

**Published:** 2026-07-13

**Authors:** Mohamed Mekhemar, Fatma E. A. Hassanein, Asmaa Abou-Bakr

**Affiliations:** 1 Clinic for Conservative Dentistry and Periodontology, School of Dental Medicine, Christian-Albrecht’s University, Kiel, Germany; 2 King Salman International University, El Tor, Egypt; 3 Galala University, Suez, Egypt

**Keywords:** toll-like receptors, innate immunity, signaling bias, MyD88, osteoimmunology, periodontal regeneration, mesenchymal stem cells

## Abstract

Periodontitis is a chronic immunoinflammatory disease characterized by site-specific destruction of the tooth-supporting tissues and marked heterogeneity in disease susceptibility, progression, and response to therapy. While dysbiotic subgingival biofilms initiate disease, microbial burden alone cannot explain the persistence of inflammation or the limited predictability of regenerative outcomes. Increasing evidence implicates innate immune dysregulation, particularly Toll-like receptor (TLR) signaling, as a central determinant of periodontal disease behavior. This narrative review synthesizes current evidence on TLR signaling in periodontal tissues, emphasizing the concept that chronic periodontitis is sustained by biased downstream signaling integration rather than uniform receptor overactivation. We discuss how persistent dominance of pro-inflammatory, MyD88-dependent pathways, coupled with insufficient engagement of regulatory and resolution-associated programs, promotes inflammatory persistence, osteoimmune imbalance, and functional impairment of periodontal stromal and stem/progenitor cells. Cell-type–specific responses to TLR activation, genetic modulation of signaling thresholds, and reciprocal interactions between innate immunity and dysbiosis are examined as key contributors to disease heterogeneity. We further explore the implications of biased TLR signaling for periodontal regeneration, proposing that regenerative failure reflects an unfavorable inflammatory signaling milieu rather than depletion of regenerative cell populations. Finally, emerging experimental strategies for interrogating and modulating TLR signaling networks—including localized immune modulation and targeted protein degradation approaches—are discussed as mechanistic research tools rather than immediate therapeutic solutions. By reframing periodontitis as a disorder of maladaptive innate immune signaling integration, this review provides a unifying conceptual framework linking dysbiosis, host-response heterogeneity, and impaired regeneration, and defines priorities for future mechanistic and translational research.

## 1 Introduction

Periodontitis is a chronic, multifactorial immunoinflammatory disease characterized by progressive destruction of the tooth-supporting tissues and remains a leading cause of tooth loss worldwide (1). Although disease initiation depends on the establishment of a dysbiotic subgingival biofilm, microbial burden alone is insufficient to explain the marked heterogeneity observed in disease susceptibility, progression rate, tissue destruction patterns, and response to therapy (2). Individuals exposed to broadly comparable microbial challenges may experience vastly different clinical outcomes, ranging from long-term periodontal stability to rapid, site-specific breakdown (3). These observations underscore the central role of host immune regulation in determining periodontal disease trajectory (3).

Innate immunity constitutes the first and most immediate layer of host defense in the periodontal environment, where tissues are continuously exposed to a dense and dynamic microbial ecosystem (4). Toll-like receptors (TLRs) are central components of this system, acting as pattern-recognition receptors that detect conserved microbial motifs as well as endogenous danger-associated signals released during tissue injury (5). The discovery of Toll-like receptors originated from studies of the Toll gene in Drosophila melanogaster, where it was initially identified as a regulator of embryonic development and later recognized for its role in antimicrobial defense (6). Subsequent identification of mammalian homologues established TLRs as central pattern-recognition receptors that bridge innate and adaptive immunity (6). Ten functional TLRs have since been identified in humans, collectively forming an integrated sensing network capable of recognizing diverse microbial and endogenous danger signals (6). Importantly, TLR expression in periodontal tissues is not limited to infiltrating immune cells but extends to gingival epithelial cells, fibroblasts, osteoblast-lineage cells, and periodontal stem and progenitor populations (7, 8). This broad cellular distribution positions TLR signaling at the intersection of microbial sensing, inflammatory amplification, osteoimmune regulation, and tissue remodeling (4).

Historically, Toll-like receptor activation in periodontitis has been viewed primarily through a pathogenic lens (9). Engagement of surface TLRs, particularly TLR2 and TLR4, by components of dysbiotic biofilms has been linked to MyD88-dependent inflammatory signaling and periodontal tissue destruction (9). This receptor-centric paradigm informed early host-modulatory concepts aimed at broadly suppressing innate immune activation (10). However, accumulating experimental and human observational evidence indicates that this interpretation is overly simplistic (4, 11).

TLR signaling outcomes are now recognized as highly context dependent (4). Receptor subtype, adaptor usage, ligand structure, signal duration, cellular differentiation state, and the surrounding inflammatory microenvironment collectively determine downstream responses (12). Under controlled or transient activation, selected TLR pathways can support immune regulation, cellular survival, and reparative processes (8, 12). In contrast, the chronically inflamed periodontal milieu favors maladaptive signaling states characterized by sustained engagement of pro-inflammatory pathways and insufficient activation of regulatory and resolution-associated programs (4).

Crucially, this context dependency is strongly cell-type specific (12). Periodontal stromal cells and stem/progenitor populations respond to TLR stimulation in ways that differ fundamentally from classical immune cells, with distinct consequences for inflammatory persistence, osteogenic potential, and immunomodulatory capacity (7, 8, 13). Rather than reflecting simple receptor overexpression or excessive ligand exposure, chronic periodontitis may therefore be sustained by a persistent qualitative bias in downstream TLR signaling toward pro-inflammatory pathways, coupled with impaired feedback regulation (4, 8). Importantly, such signaling bias does not imply permanent rewiring of innate immune pathways, but rather a dynamically maintained inflammatory state shaped by persistent microenvironmental cues (14).

Despite growing recognition of these principles, much of the periodontal literature continues to focus on TLR expression levels or ligand–receptor interactions, often overlooking the qualitative nature of downstream signaling integration (15, 16). This emphasis obscures a critical conceptual distinction: whether chronic periodontitis is driven by generalized overactivation of innate immune receptors or by maladaptive skewing of intracellular signaling networks that determine inflammatory persistence and tissue fate. Resolving this distinction is essential for understanding site-specific disease activity, heterogeneous treatment responses, and the limited predictability of regenerative outcomes.

In this narrative review, current evidence on Toll-like receptor signaling in periodontal tissues is synthesized with particular emphasis on context-dependent and cell-type–specific signaling bias. The contribution of TLRs to periodontal pathogenesis, the modulatory role of host genetic variation, and the consequences of persistent innate immune activation for periodontal regeneration are examined. In addition, emerging experimental strategies designed to dissect causal signaling nodes within complex TLR networks, including targeted protein degradation approaches, are discussed as mechanistic research tools rather than immediate therapeutic solutions. By reframing periodontitis as a disorder sustained by biased innate immune signaling rather than uniform receptor overactivation, this review aims to provide a coherent framework for understanding disease heterogeneity, inflammatory persistence, and impaired regenerative capacity.

## 2 Literature identification and scope of the review

This narrative review is based on a comprehensive appraisal of the literature addressing Toll-like receptor signaling in periodontitis and related periodontal inflammatory conditions. Relevant publications were identified through structured searches of PubMed/MEDLINE and Web of Science, with a primary focus on peer-reviewed articles published in English. Emphasis was placed on recent literature to reflect current mechanistic understanding, while seminal earlier studies were included where necessary to provide historical and conceptual context.

Search strategies employed combinations of key terms including “periodontitis,” “Toll-like receptors,” “innate immunity,” “TLR signaling,” “MyD88,” “TRIF,” “osteoinflammation,” “immune tolerance,” and “periodontal regeneration.” Experimental *in vitro* and *in vivo* studies, translational research, and human observational investigations were considered to capture both mechanistic insight and clinically relevant associations. Reference lists of selected key articles were manually screened to identify additional publications of relevance.

Given the hypothesis-driven and conceptual nature of this review, study selection was guided by scientific relevance to signaling integration, pathway bias, and cell-type specificity rather than by formal systematic inclusion or exclusion criteria. Accordingly, the review prioritizes qualitative interpretation of downstream signaling dynamics and functional outcomes over exhaustive cataloguing of receptor expression studies or descriptive correlations.

## 3 Toll-like receptors in periodontal tissues: molecular architecture and signaling pathways

Toll-like receptors constitute a conserved family of pattern-recognition receptors that play a central role in innate immune surveillance by detecting microbial-associated and damage-associated molecular patterns (14). In humans, ten functional TLRs (TLR1–TLR10) have been identified, each sharing a common structural organization consisting of an extracellular leucine-rich repeat domain responsible for ligand recognition, a single transmembrane region, and a cytoplasmic Toll/interleukin-1 receptor domain that initiates intracellular signaling (14). Despite this conserved architecture, individual TLRs differ markedly in ligand specificity, cellular localization, adaptor recruitment, and signaling output (14). The principal characteristics of human TLRs relevant to periodontal biology, including their cellular localization, ligands, signaling adaptors, and reported periodontal relevance, are summarized in **Table 1**.

**Table 1:** Human toll-like receptors relevant to periodontal biology: cellular localization, principal ligands, signaling adaptors, and reported relevance in periodontal tissues.

TLR	Cellular localization	Principal ligands	Major adaptor(s)	Reported relevance in periodontal tissues
TLR1	Cell surface	Triacylated bacterial lipopeptides (heterodimer with TLR2)	MyD88	Contributes to recognition of bacterial components within the subgingival biofilm
TLR2	Cell surface	Bacterial lipoproteins, peptidoglycan, lipoteichoic acid, atypical periodontal pathogen ligands	MyD88	Major contributor to inflammatory signaling induced by periodontal pathogens
TLR3	Endosome	Double-stranded RNA	TRIF	Associated with immunomodulatory, stress-adaptive, and regenerative responses in periodontal stromal and progenitor cells
TLR4	Cell surface and endosome	Lipopolysaccharide (LPS), endogenous danger-associated molecular patterns (DAMPs)	MyD88, TRIF	Central mediator of inflammatory amplification, osteoimmune regulation, and tissue destruction
TLR5	Cell surface	Flagellin	MyD88	May contribute to epithelial immune surveillance and antimicrobial defense
TLR6	Cell surface	Diacylated bacterial lipopeptides (heterodimer with TLR2)	MyD88	Participates in cooperative recognition of bacterial ligands
TLR7	Endosome	Single-stranded RNA	MyD88	Emerging evidence suggests involvement in immune-cell activation within inflamed periodontal tissues
TLR8	Endosome	Single-stranded RNA	MyD88	May contribute to inflammatory cytokine responses and innate immune activation
TLR9	Endosome	Unmethylated CpG DNA	MyD88	Implicated in host susceptibility, inflammatory regulation, and osteoimmune responses
TLR10	Cell surface and endosome	Ligands incompletely defined	Not fully characterized	Potential immunomodulatory functions; periodontal relevance remains incompletely understood

TLR2 and TLR4 remain the most extensively studied receptors in periodontitis and therefore constitute the primary focus of this review. The periodontal roles of several other TLR family members continue to emerge and remain incompletely characterized.

In periodontal tissues, TLR expression extends beyond infiltrating immune cells to include gingival epithelial cells, fibroblasts, osteoblast-lineage cells, and mesenchymal stem and progenitor populations (7, 8). Surface-expressed TLRs, including TLR1, TLR2, TLR4, TLR5, and TLR6, primarily recognize bacterial components abundant within the subgingival biofilm (7). Endosomal TLRs, notably TLR3, TLR7, TLR8, and TLR9, sense nucleic acids derived from viruses, bacteria, or damaged host cells, particularly under conditions of epithelial disruption and tissue injury (7).

### 3.1 Canonical TLR signaling pathways: MyD88- and TRIF-dependent routes

Upon ligand binding, TLRs undergo dimerization and recruit adaptor proteins to their cytoplasmic domains, initiating intracellular signaling cascades (17). Two principal signaling routes mediate these responses: the MyD88-dependent pathway and the TRIF-dependent pathway (18). The balance between these routes is a key determinant of downstream biological outcome (17).

The MyD88-dependent pathway is engaged by all TLRs except TLR3 and mediates rapid inflammatory responses (17). Recruitment of MyD88 leads to activation of interleukin-1 receptor–associated kinases and tumor necrosis factor receptor–associated factor 6, culminating in activation of NF-κB and MAPK pathways and induction of pro-inflammatory cytokines, chemokines, and matrix-degrading enzymes (19). In periodontal tissues, sustained MyD88-dependent signaling downstream of TLR2 and TLR4 has been consistently associated with inflammatory amplification, osteoclastogenic signaling, and connective tissue breakdown (20–22).

In contrast, the TRIF-dependent pathway is engaged by TLR3 and by TLR4 following receptor endocytosis (14). TRIF signaling activates TANK-binding kinase 1 and IKKϵ, leading to phosphorylation of interferon regulatory factors and induction of type I interferons and interferon-stimulated genes (14). Beyond antiviral defense, TRIF-associated signaling has been linked to immunomodulatory, stress-adaptive, and survival-promoting programs in selected cellular contexts (23). Importantly, TRIF signaling can exert either regulatory or inflammatory effects depending on ligand characteristics, signal duration, and cell type (14, 24).

### 3.2 Additional TLR family members in periodontal immunity

Although TLR2 and TLR4 represent the most extensively investigated receptors in periodontitis (25) and therefore receive primary emphasis throughout this review, accumulating evidence indicates that several additional TLR family members contribute to periodontal immune regulation (26). These receptors recognize distinct classes of microbial and endogenous ligands, engage different signaling adaptors, and may influence inflammatory persistence, tissue destruction, and regenerative responses in a context-dependent manner (26).

Among endosomal receptors, TLR3 has attracted particular interest because of its unique reliance on TRIF-dependent signaling (8). TLR3 recognizes double-stranded RNA and has been implicated in immunomodulatory, stress-adaptive, and regenerative responses in several stromal cell populations (8). In periodontal tissues, TLR3 is expressed by mesenchymal stem and progenitor cells, where controlled activation has been associated with enhanced survival, altered differentiation trajectories, and modulation of immunoregulatory functions (27). These observations suggest that TLR3 may contribute to signaling programs distinct from the predominantly pro-inflammatory responses associated with sustained TLR2- and TLR4-mediated activation (27).

TLR5 recognizes bacterial flagellin and is primarily expressed on epithelial and innate immune cells (28). Through its role in sensing motile bacteria, TLR5 contributes to maintenance of mucosal barrier function and early antimicrobial defense (29). Although its role in periodontitis has been investigated less extensively than that of TLR2 or TLR4, evidence suggests that TLR5 participates in epithelial immune surveillance and may influence inflammatory responses within the periodontal environment (30).

Additional endosomal receptors, including TLR7 and TLR8, recognize single-stranded RNA and contribute to antiviral and inflammatory signaling (31). Increased expression of these receptors has been reported in inflamed periodontal tissues (30, 32), and emerging evidence suggests that they may participate in immune-cell activation and inflammatory amplification (30, 32). However, their precise contribution to periodontal disease progression remains incompletely defined and requires further investigation.

TLR9 detects unmethylated CpG motifs present in bacterial and viral DNA and has been implicated in both innate immune activation and osteoimmune regulation (33). In periodontal research, TLR9 has attracted interest because of its potential role in host susceptibility, inflammatory signaling, and regulation of bone remodeling (34). Genetic association studies have further suggested that variation within TLR9-related pathways may influence individual responses to periodontal challenge, although findings remain heterogeneous across populations (35, 36).

Collectively, these observations indicate that periodontal immunity is shaped by the coordinated activity of multiple TLR family members rather than by isolated receptor pathways (25). Nevertheless, current evidence supporting cell-type–specific signaling bias, inflammatory persistence, and regenerative modulation remains strongest for TLR2- and TLR4-associated networks (25). This evidence base explains the primary focus on these receptors throughout the present review while recognizing that additional TLR family members may contribute to periodontal homeostasis and disease in context-dependent ways.

### 3.3 Regulatory checkpoints and negative feedback in TLR signaling

TLR signaling is subject to multilayered regulation by intracellular negative feedback mechanisms that limit excessive or prolonged activation (37). Molecules such as Toll-interacting protein (38), A20 (39), suppressor of cytokine signaling proteins (40), and IRAK-M (41) act at different levels of the signaling cascade to dampen inflammatory output and promote resolution. Under physiological conditions, these mechanisms ensure proportional immune responses compatible with tissue homeostasis (42).

In chronic inflammatory settings such as periodontitis, accumulating evidence suggests that regulatory checkpoints may be attenuated or dysfunctional (43). Persistent microbial stimulation, oxidative stress, and sustained cytokine exposure can impair feedback control, leading to resistance to tolerance induction and prolonged pathway activation (44). Importantly, this loss of regulation does not merely increase signaling magnitude but qualitatively reshapes downstream integration, favoring inflammatory persistence over resolution (14).

### 3.4 TLR signaling in the periodontal context

The periodontal microenvironment presents unique challenges for innate immune regulation (4). Continuous microbial exposure under conditions that do not permit complete pathogen elimination requires precise modulation of inflammatory intensity and duration (4). TLR signaling in this setting functions as a regulatory system rather than a simple on–off switch (14).

Under homeostatic conditions, low-level TLR engagement contributes to immune surveillance and barrier integrity without inducing tissue damage (45). During dysbiosis-associated inflammation, however, sustained activation of surface TLRs—particularly TLR2 and TLR4—can overwhelm regulatory mechanisms and drive persistent MyD88-dominant signaling (2). This shift reflects not only increased receptor engagement but a qualitative bias in downstream pathway utilization, setting the stage for inflammatory persistence and tissue destruction (2).

## 4 Physiological versus pathological TLR activation in periodontal homeostasis

Periodontal tissues are continuously exposed to a dense and diverse microbial community, yet under physiological conditions they maintain a state of controlled immune surveillance without overt tissue destruction (2). This equilibrium is achieved through finely regulated innate immune signaling, in which Toll-like receptors play a central modulatory role (11). Importantly, TLR activation in health does not equate to chronic inflammation but instead supports barrier integrity, microbial containment, and immune readiness (46).

Under homeostatic conditions, low-level or intermittent engagement of TLRs by commensal microorganisms induces restrained signaling responses (46). Gingival epithelial cells and stromal cells respond to such stimulation by producing antimicrobial peptides and regulatory cytokines while avoiding excessive activation of pro-inflammatory transcriptional programs (47). In this context, TLR signaling functions as a calibrating mechanism that enables periodontal tissues to coexist with the resident microbiota while preserving structural integrity (47).

The transition from periodontal health to disease is marked by a qualitative shift in TLR signaling dynamics rather than by a simple increase in receptor engagement (4, 48). Dysbiosis-associated inflammation exposes periodontal tissues to persistent and complex microbial stimuli that favor sustained activation of surface TLRs, particularly TLR2 and TLR4 (49). Unlike transient physiological signaling, chronic engagement overwhelms regulatory checkpoints and leads to prolonged MyD88-dependent pathway activation (49).This shift results in continuous NF-κB and MAPK signaling, excessive production of pro-inflammatory cytokines, and amplification of tissue-destructive processes (49).

A defining feature of pathological TLR activation in periodontitis is resistance to tolerance and impaired resolution (50–52). In many tissues, repeated TLR stimulation induces hyporesponsiveness through feedback inhibition, thereby preventing uncontrolled inflammation (53). In the chronically inflamed periodontal environment, however, tolerance mechanisms appear insufficient or dysfunctional (50, 51). Persistent microbial stimulation, combined with oxidative stress and a cytokine-rich milieu, may disrupt negative feedback loops and sustain inflammatory signaling even in the absence of escalating microbial burden (50, 51).

Importantly, resolution of inflammation should not be viewed merely as passive termination of TLR signaling. Resolution is now recognized as an active biological process orchestrated by specialized pro-resolving mediators (SPMs), including resolvins, lipoxins, protectins, and maresins (54, 55). These mediators promote cessation of neutrophil recruitment, clearance of inflammatory cells, restoration of tissue homeostasis, and tissue repair (55). Experimental and clinical periodontal studies suggest that impaired engagement of resolution-associated pathways may contribute to persistent inflammation despite reduction of microbial burden (48). Consequently, chronic periodontal inflammation may reflect both excessive inflammatory signaling and inadequate activation of endogenous resolution programs.

**Figure 1:**
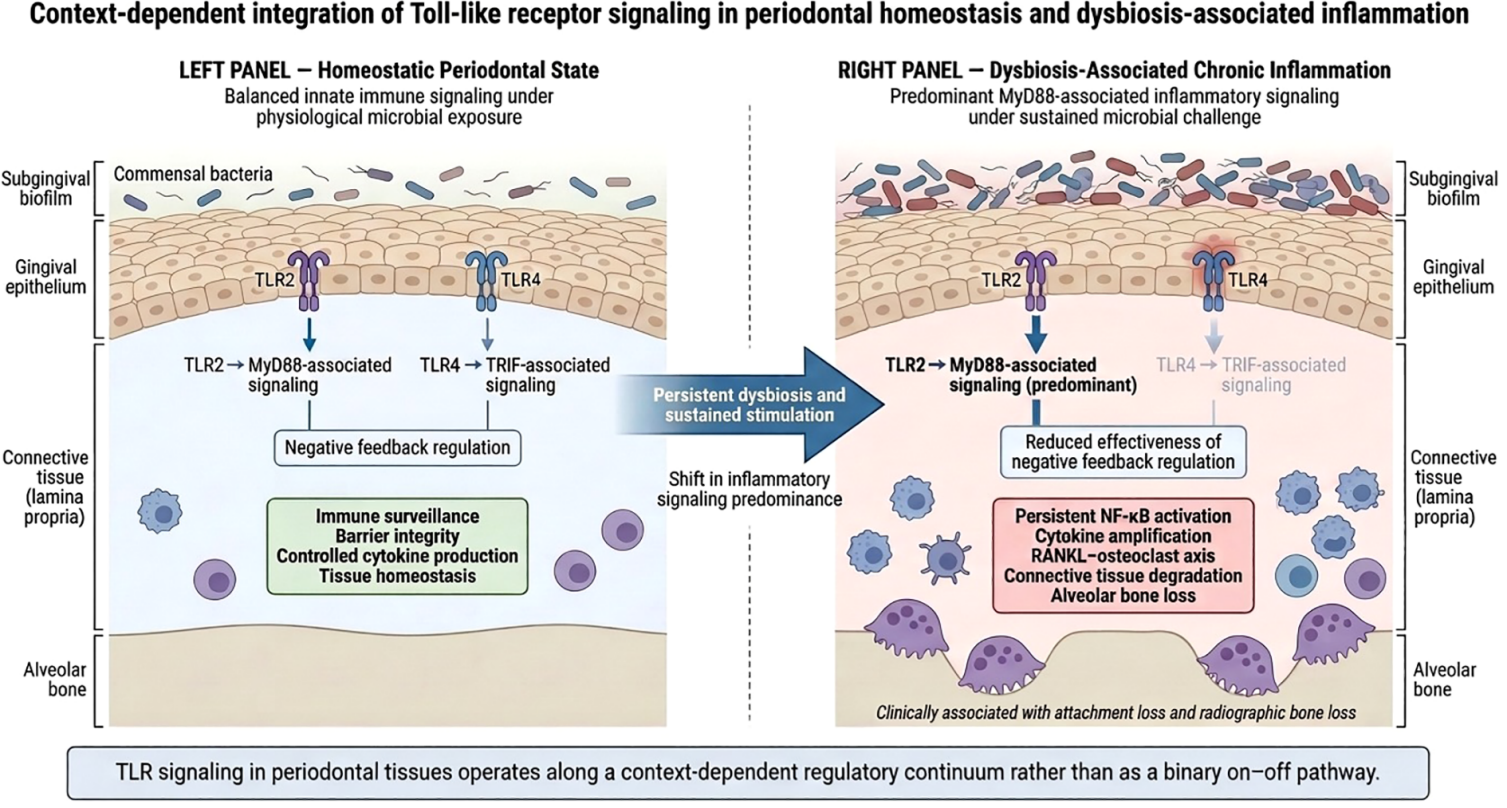
Context-dependent integration of toll-like receptor signaling in periodontal homeostasis and dysbiosis-associated inflammation.

Loss of signaling restraint has important consequences for tissue integrity. Sustained TLR-driven cytokine release promotes recruitment and activation of immune cells, enhances expression of matrix metalloproteinases, and shifts osteoimmune balance toward osteoclastogenesis (4, 48). At the same time, chronic inflammatory signaling interferes with reparative processes by impairing stromal cell function and suppressing regenerative pathways (41). Thus, pathological TLR activation not only drives tissue destruction (56) but may also interfere with mechanisms required for resolution and repair (6), including specialized pro-resolving mediator pathways, resolution-associated macrophage functions, osteogenic signaling programs, and tissue-remodeling processes necessary for restoration of periodontal homeostasis (3). Physiological and pathological TLR activation therefore cannot be distinguished solely by receptor expression levels or ligand availability (56). Instead, they differ in the duration, intensity, and integration of downstream signaling responses (56). While homeostatic signaling is transient, spatially restricted, and subject to effective feedback control, pathological signaling is persistent, spatially diffuse, and biased toward pro-inflammatory pathways (56). This distinction underscores the importance of signaling quality rather than signaling presence in determining periodontal tissue fate (**Figure 1**).

Under physiological conditions (left panel), low-level engagement of TLR2 and TLR4 by commensal microbial stimuli is associated with balanced MyD88- and TRIF-related signaling constrained by effective negative feedback regulation. This controlled signaling integration supports immune surveillance, epithelial barrier integrity, and regulated cytokine production, thereby maintaining periodontal tissue homeostasis. Under dysbiosis-associated chronic inflammation (right panel), sustained microbial stimulation is associated with predominant MyD88-linked inflammatory signaling and reduced effectiveness of negative feedback regulation. This shift correlates with persistent NF-κB activation, cytokine amplification, RANKL-mediated osteoclastogenesis, connective tissue degradation, and alveolar bone loss, clinically manifesting as attachment loss and radiographic bone destruction. The schematic illustrates that TLR signaling in periodontal tissues operates along a context-dependent regulatory continuum rather than as a binary on–off pathway.

Taken together, periodontal homeostasis depends on the ability of TLR signaling networks to balance immune defense with tissue preservation (26). Periodontitis emerges when this balance is disrupted and innate immune signaling becomes locked in a maladaptive state that favors inflammatory persistence over resolution (26). Recognizing this transition as a disorder of signaling regulation rather than excessive immune activation provides a conceptual foundation for understanding disease chronicity and sets the stage for examining cell-type–specific dysregulation in periodontal tissues.

## 5 Cell-type–specific dysregulation of TLR signaling in periodontitis

A defining feature of Toll-like receptor signaling in periodontitis is its pronounced cell-type specificity (7). Periodontal tissues comprise a heterogeneous assembly of epithelial, stromal, immune, and progenitor cell populations, each characterized by distinct signaling thresholds, adaptor usage, and feedback regulation (7, 8). Consequently, dysregulated TLR responses in periodontitis are spatially and functionally compartmentalized rather than uniform across inflamed tissues (47). This cellular heterogeneity provides an important framework for understanding site-specific disease activity and variability in clinical outcomes. The principal Toll-like receptor subsets, signaling characteristics, and functional consequences across major periodontal cell populations are summarized in **Table 2**.

**Table 2:** Cell-type–specific TLR signaling characteristics in periodontal tissues.

Cell type	Predominantly studied TLRs	Signaling bias in periodontitis	Functional consequences	Contribution to disease
Gingival epithelial cells	TLR2, TLR4, TLR5, TLR9	Sustained NF-κB activation; reduced tolerance	Cytokine production; leukocyte recruitment	Persistent inflammatory infiltration
Gingival fibroblasts	TLR2, TLR3, TLR4, TLR9	Prolonged cytokine/MMP production	ECM degradation; impaired remodeling	Connective tissue breakdown
Macrophages	TLR2, TLR4, TLR7, TLR8, TLR9	Pro-inflammatory polarization	TNF-α, IL-1β production; osteoclastogenic signaling	Osteoimmune amplification
Neutrophils	TLR2, TLR4	Excessive activation	ROS and protease release	Collateral tissue damage
Osteoblast-lineage cells	TLR2, TLR4, TLR9	RANKL/OPG imbalance	Enhanced osteoclastogenesis	Bone resorption
PDL stem/progenitor cells	TLR1, TLR2, TLR3, TLR4, TLR5, TLR6, TLR9	Chronic inflammatory bias with relative predominance of MyD88-associated signaling	Impaired osteogenic differentiation; reduced immunomodulation	Regeneration failure

The listed receptors represent the predominantly reported or functionally studied TLR family members in each cell population and should not be interpreted as an exhaustive expression profile.

### 5.1 Gingival epithelial cells

Gingival epithelial cells form the primary barrier between the host and the subgingival biofilm and represent one of the earliest cellular interfaces for microbial recognition (57). Under physiological conditions, epithelial TLR signaling supports barrier integrity and antimicrobial defense through restrained activation of innate immune pathways (57). In periodontitis, however, epithelial cells exhibit heightened responsiveness to microbial ligands, particularly through TLR2- and TLR4-mediated signaling (48).

Chronic exposure to dysbiotic biofilms leads to sustained activation of epithelial NF-κB signaling, resulting in increased production of pro-inflammatory cytokines and chemokines that promote leukocyte recruitment (48). Importantly, epithelial cells derived from periodontitis lesions often display impaired attenuation of inflammatory signaling upon repeated stimulation, suggesting a loss of tolerance mechanisms that normally limit inflammatory escalation (51). This altered epithelial responsiveness contributes to persistent inflammatory cell infiltration and facilitates deeper microbial penetration, thereby reinforcing local inflammatory circuits (58).

### 5.2 Gingival fibroblasts and stromal cells

Gingival fibroblasts, once considered passive structural elements, are now recognized as active participants in innate immune regulation (59, 60). These cells express multiple TLRs and respond directly to microbial and inflammatory stimuli (60). In the context of periodontitis, fibroblasts isolated from inflamed tissues demonstrate aberrant TLR signaling characterized by sustained cytokine and matrix metalloproteinase production (61).

Chronic activation of TLR2 and TLR4 in gingival fibroblasts promotes expression of interleukin-6, interleukin-8, and other mediators that perpetuate inflammatory cell recruitment and extracellular matrix degradation (48). Notably, fibroblasts exhibit limited capacity to downregulate inflammatory signaling once activated (62), effectively functioning as local amplifiers of inflammation. Prolonged TLR-driven signaling also alters fibroblast–matrix interactions, contributing to dysregulated tissue remodeling and loss of connective tissue architecture (62).

### 5.3 Immune cells within periodontal lesions

Immune cells infiltrating periodontal lesions, including macrophages, neutrophils, and dendritic cells, display heightened TLR responsiveness in chronic disease (3). Persistent engagement of TLR2 and TLR4 in these populations sustains pro-inflammatory cytokine production and reinforces osteoclastogenic signaling (63). In macrophages, chronic TLR stimulation favors polarization toward pro-inflammatory phenotypes while impairing the transition to resolution-associated states (64).

Neutrophils, which are abundant in periodontal lesions, are similarly influenced by dysregulated TLR signaling (65). Excessive activation enhances reactive oxygen species production and protease release, contributing to collateral tissue damage (66). Importantly, mediators released by immune cells feed back onto stromal and epithelial cells, creating a self-perpetuating inflammatory network within the periodontal microenvironment (48).

### 5.4 Osteoimmune interactions and bone remodeling

TLR signaling exerts profound effects on alveolar bone homeostasis through its influence on osteoimmune interactions (67). Osteoblast-lineage cells and osteoclast precursors express functional TLRs and respond directly to microbial stimuli (67). Sustained TLR2- and TLR4-mediated signaling enhances expression of receptor activator of nuclear factor κB ligand while suppressing osteoprotegerin, thereby shifting the balance toward osteoclastogenesis and bone resorption (67).

In addition to direct effects on bone cells, inflammatory cytokines produced downstream of TLR activation further amplify osteoclast differentiation and activity (67). This integrated osteoimmune response illustrates how dysregulated innate immune signaling translates microbial recognition into irreversible structural damage (4). Importantly, once established, these pathways may remain active even after reduction of microbial load, highlighting the relative autonomy of inflammatory signaling circuits in driving disease progression (3).

### 5.5 Implications of cellular signaling bias

**Figure 2:**
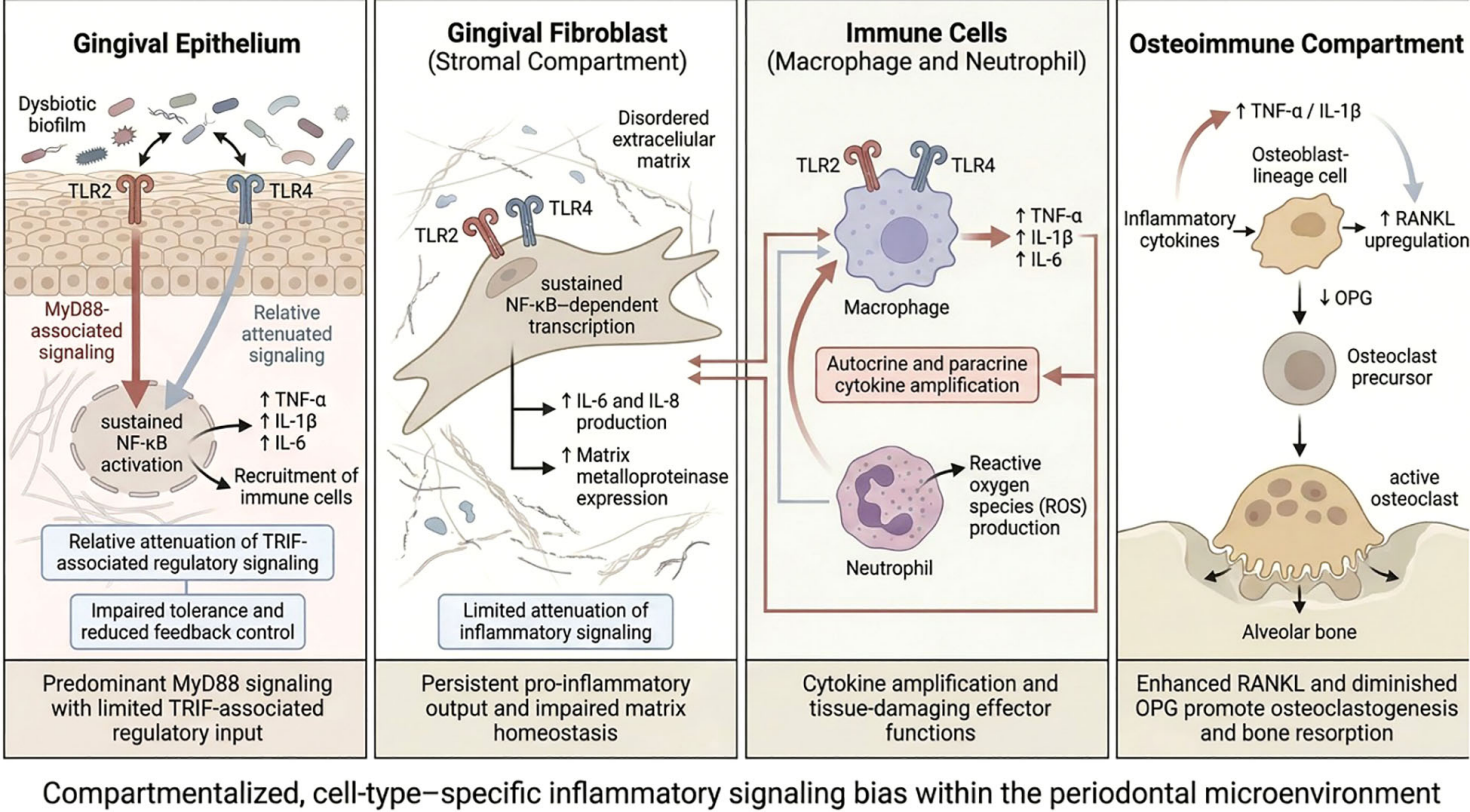
Compartmentalized, cell-type–specific integration of toll-like receptor signaling in periodontitis.

Collectively, these observations indicate that periodontitis is characterized by cell-type–specific loss of signaling balance rather than uniform innate immune hyperactivation (48). Epithelial cells, fibroblasts, immune cells, and bone-associated populations each contribute distinct but interconnected components to the inflammatory milieu (68). The convergence of these dysregulated responses creates localized microenvironments in which pro-inflammatory TLR signaling dominates, while regulatory and resolution-associated pathways remain insufficiently engaged (69). This compartmentalized signaling bias provides a mechanistic explanation for the heterogeneity observed in periodontal disease, including site-specific activity and variable response to therapy (70). It also reinforces the concept that effective intervention requires restoration of signaling balance within specific cellular niches rather than global suppression of innate immune pathways (4, 71) (**Figure 2**).

Sustained engagement of TLR2 and TLR4 under dysbiosis-associated conditions results in cell-type–specific inflammatory signaling bias within the periodontal microenvironment. In gingival epithelial cells, predominant MyD88-associated signaling with relative attenuation of TRIF-associated regulatory pathways promotes sustained NF-κB activation, cytokine production, and immune cell recruitment. Gingival fibroblasts exhibit persistent NF-κB–dependent transcription, increased interleukin-6 and interleukin-8 production, and enhanced matrix metalloproteinase expression, contributing to extracellular matrix disruption and impaired matrix homeostasis. In infiltrating immune cells, including macrophages and neutrophils, sustained TLR signaling amplifies tumor necrosis factor-α, interleukin-1β, and interleukin-6 production, promotes reactive oxygen species generation and protease release, and reinforces autocrine and paracrine inflammatory loops. Within the osteoimmune compartment, inflammatory cytokines increase RANKL expression and reduce osteoprotegerin levels in osteoblast-lineage cells, thereby promoting osteoclast differentiation and alveolar bone resorption. Collectively, these compartmentalized responses generate localized inflammatory microenvironments characterized by persistent MyD88-dominant signaling bias and insufficient regulatory counterbalance.

## 6 TLR-driven dysbiosis and immune evasion in periodontitis

The interaction between Toll-like receptor signaling and the subgingival microbiota is fundamentally bidirectional (2). While TLRs function as primary sensors of microbial challenge, the inflammatory milieu shaped by their downstream signaling also exerts selective pressure on the microbial ecosystem (2). In periodontitis, dysregulated TLR signaling contributes not only to inflammatory tissue destruction but also to stabilization of a dysbiotic biofilm, thereby reinforcing disease chronicity (72).

Under physiological conditions (73), restrained TLR activation supports microbial containment without imposing strong selective pressure on commensal communities. In contrast, persistent activation of TLR2- and TLR4-dependent pathways establishes a chronically inflamed periodontal microenvironment characterized by elevated cytokine levels, increased availability of tissue breakdown products, and altered nutrient gradients (48). These conditions favor the expansion of proteolytic, inflammation-adapted species and promote ecological shifts toward a dysbiotic biofilm capable of thriving under inflammatory stress.

Keystone periodontal pathogens have evolved strategies to exploit this inflammatory niche by actively manipulating TLR signaling (20). Porphyromonas gingivalis is the most extensively studied example of such immune subversion (20, 74). Structural heterogeneity of its lipopolysaccharide lipid A moieties allows *P. gingivalis* to attenuate or antagonize TLR4 signaling while simultaneously engaging TLR2-dependent pathways (75, 76). This selective pattern of receptor engagement suppresses effective bactericidal responses while sustaining low-grade inflammation, thereby permitting microbial persistence without triggering robust clearance (74).

Beyond receptor-level effects, dysbiotic bacteria influence downstream signaling integration (77). Preferential activation of TLR2 has been associated with inflammatory responses that lack efficient resolution while inducing a state of partial tolerance (78), in which innate immune cells remain refractory to strong antimicrobial activation yet continue to produce pro-inflammatory mediators. This paradoxical state supports immune evasion and sustains tissue-damaging inflammation (79).

Macrophages represent key cellular targets of this immune manipulation (80). Dysbiotic species exploit TLR-mediated pathways to alter macrophage polarization and intracellular survival mechanisms, facilitating bacterial persistence within host cells (80). Concurrently, chronic TLR engagement enhances production of inflammatory cytokines that amplify connective tissue breakdown and further modify the microbial habitat (81). Thus, immune evasion and immune activation coexist and reinforce each other rather than functioning as opposing processes (78).

Importantly, periodontal dysbiosis reflects the collective activity of a microbial consortium rather than the action of a single pathogen (82). Species such as Fusobacterium nucleatum, Tannerella forsythia, and Treponema denticola interact with TLR signaling networks to promote epithelial barrier disruption, inflammatory amplification, and tissue invasion (30, 83). While the contribution of endosomal TLR engagement in periodontal tissues remains incompletely defined (4), epithelial damage and host cell death may increase exposure to microbial and endogenous nucleic acids (4, 14), potentially amplifying innate immune signaling through additional pathways (84).

**Figure 3:**
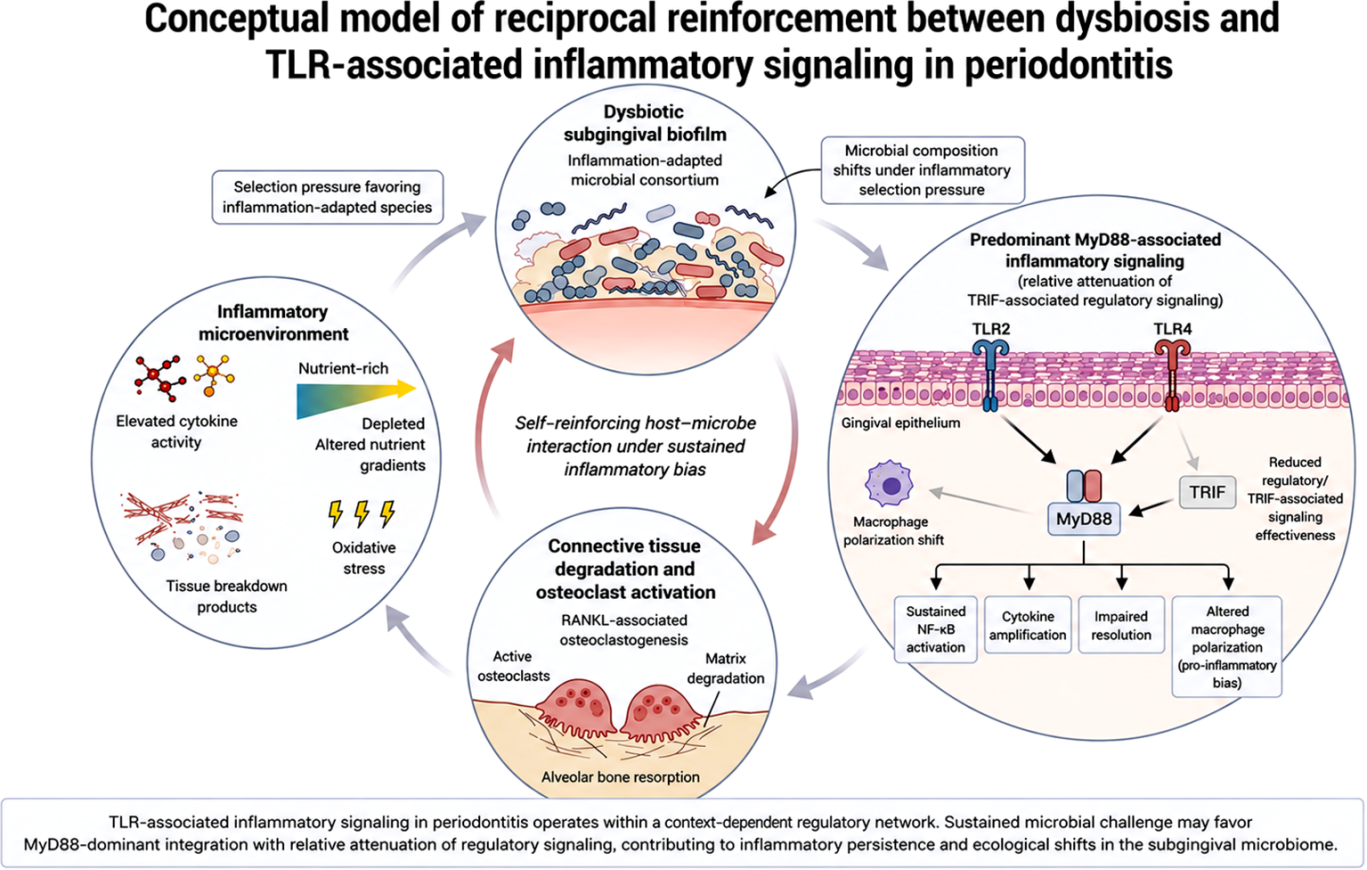
Conceptual model of reciprocal reinforcement between dysbiosis and TLR-associated inflammatory signaling in periodontitis.

TLR-driven dysbiosis is therefore not merely a consequence of microbial overgrowth but reflects an altered host–microbe equilibrium maintained by maladaptive innate immune signaling (85). Even after mechanical reduction of biofilm load, residual inflammatory signaling can preserve ecological conditions that favor re-establishment of dysbiosis (2, 86). This phenomenon provides a biological explanation for disease recurrence and site-specific persistence despite adequate antimicrobial therapy (2, 86) (**Figure 3**).

Sustained microbial challenge within the subgingival biofilm may favor a predominance of MyD88-associated inflammatory signaling downstream of surface Toll-like receptors, particularly TLR2 and TLR4, with relative attenuation of TRIF-associated regulatory signaling pathways. This signaling bias is associated with persistent NF-κB activation, cytokine amplification, impaired resolution, and altered macrophage polarization. The resulting inflammatory microenvironment—characterized by elevated cytokine levels, tissue breakdown products, oxidative stress, and altered nutrient gradients—creates ecological selection pressure favoring inflammation-adapted microbial communities. Connective tissue degradation and RANKL-associated osteoclast activation contribute to alveolar bone resorption, further modifying the local habitat. Together, these processes illustrate a context-dependent, self-reinforcing host–microbe interaction rather than a linear or deterministic cascade.

## 7 Genetic modulation of TLR signaling thresholds and host-response heterogeneity

Pronounced interindividual variability in periodontal disease susceptibility, progression, and treatment response (87) suggests that host-intrinsic factors modulate innate immune responsiveness (87, 88). Genetic variation within Toll-like receptor signaling pathways has therefore been investigated as a potential contributor to periodontal disease heterogeneity (89). Rather than acting as deterministic drivers of disease, available evidence indicates that such variants primarily function as modifiers of inflammatory signaling thresholds and persistence (90).

Single-nucleotide polymorphisms affecting TLR2, TLR4, TLR9, and key adaptor molecules have been associated with altered cytokine responses following microbial stimulation (91). Many of these variants influence receptor conformation, ligand interaction, receptor trafficking, or downstream signaling efficiency, thereby tuning the magnitude, kinetics, and duration of inflammatory responses (91–93). In the setting of chronic microbial exposure, even modest shifts in signaling thresholds may bias immune responses toward prolonged activation or impaired resolution (42).

TLR4 polymorphisms, such as Asp299Gly and Thr399Ile (92–94), have been most extensively studied in relation to periodontitis. Early reports suggested that some variants attenuate lipopolysaccharide responsiveness (95) and therefore affect the susceptibility to periodontal breakdown. Subsequent functional and population-based studies, however, have yielded heterogeneous results, with some variants associated with reduced inflammatory destruction (96) and other variants showing higher susceptibility to inflammation (92). These divergent findings underscore the context dependency of genetic effects and highlight the influence of microbial composition, co-receptor availability, and environmental modifiers on functional outcomes (4).

Variants in TLR2 and TLR1 have also been linked to altered responsiveness to bacterial lipoproteins and changes in downstream cytokine production (97). Such polymorphisms may shift signaling balance toward exaggerated or prolonged MyD88-dependent activation, particularly in environments characterized by persistent microbial challenge. Although reported effect sizes are generally modest, these variants may contribute cumulatively to inflammatory burden over time (98), especially when combined with established risk factors such as smoking, diabetes mellitus, or other systemic inflammatory conditions (99).

Evidence regarding TLR9 polymorphisms in periodontitis remains less consistent (35). Promoter variants influencing TLR9 expression have been associated with altered immune responses in several inflammatory disorders (100), but direct functional validation in periodontal tissues is limited. Nonetheless, nucleic acid sensing through endosomal TLRs may contribute to inflammatory amplification in advanced periodontal lesions, where tissue breakdown and host cell death increase exposure to endogenous danger-associated nucleic acids (101).

Importantly, genetic variation does not appear to determine disease occurrence in isolation (90). Instead, it modulates the likelihood that innate immune responses will resolve appropriately or become locked in maladaptive states (90). From this perspective, TLR polymorphisms are best viewed as factors that influence signaling bias (102) rather than as binary risk determinants. This interpretation aligns with the observed heterogeneity of periodontal disease expression and the limited predictive value of individual genetic markers in clinical practice (103).

## 8 Consequences of TLR signaling bias for periodontal regeneration

Successful periodontal regeneration requires more than effective microbial control; it depends on restoration of a tissue microenvironment permissive for repair, osteogenesis, and re-establishment of functional attachment (104). Increasing evidence indicates that persistent Toll-like receptor–driven inflammation compromises this environment by sustaining an osteoimmune imbalance that is fundamentally incompatible with regeneration (67, 105). Within this framework, regenerative failure in periodontitis reflects prolonged innate immune signaling bias rather than an intrinsic deficiency of regenerative cell populations (106). This functional impairment may be further reinforced by emerging concept of ‘inflammatory memory’ or ‘trained immunity’ in non-immune cells, whereby prior exposure to inflammatory stimuli can result in long-lasting epigenetic reprogramming, thereby fixing cells in a persistent, hypo-responsive, or dysfunctional state long after the initial insult has been cleared.

Although persistent TLR-associated inflammatory signaling represents a major contributor to regenerative impairment (69), periodontal regeneration is influenced by multiple interconnected host-response pathways (106). Defective activation of specialized pro-resolving mediator (SPM) networks (107, 108), osteoimmune dysregulation (109), altered angiogenic responses (110), and persistent stromal reprogramming (111) may also contribute to impaired tissue repair. Accordingly, regenerative failure is best viewed as a multifactorial consequence of an unfavorable microenvironment rather than the result of any single signaling pathway. Within this broader host-response network, persistent TLR-associated signaling remains a major mechanistic contributor to regenerative impairment and will therefore be considered in greater detail below.

Chronic activation of TLR2- and TLR4-dependent pathways (26, 105) exerts sustained effects on both immune and stromal compartments, extending beyond overt tissue destruction. Pro-inflammatory cytokines such as tumor necrosis factor-α, interleukin-1β, and interleukin-6, produced downstream of persistent MyD88-dependent signaling, directly inhibit osteogenic differentiation and extracellular matrix synthesis (112). At the same time, these mediators promote expression of receptor activator of nuclear factor κB ligand while suppressing osteoprotegerin, thereby reinforcing osteoclastogenesis and preventing restoration of alveolar bone architecture (68).

The inflammatory milieu shaped by maladaptive TLR signaling also alters the functional behavior of resident stromal cells (7, 13). Gingival fibroblasts and periodontal ligament cells exposed to chronic inflammatory cues exhibit reduced anabolic activity, altered matrix turnover, and impaired capacity to support organized tissue regeneration (61, 113). These changes reflect active reprogramming of cellular function driven by persistent innate immune signaling rather than passive consequences of tissue damage (114).

Periodontal stem and progenitor cells (7), which are central mediators of regeneration, are particularly sensitive to microenvironmental cues. Sustained exposure to TLR-driven cytokines interferes with osteogenic differentiation, promotes cellular stress responses, and limits the capacity of these cells to contribute effectively to repair (7). Available evidence suggests that functional impairment is more prominent than outright depletion of stem and progenitor populations, indicating that regenerative potential is constrained by the surrounding signaling environment (115).

Clinical observations are consistent with this mechanistic framework (116). Regenerative procedures—including guided tissue regeneration, enamel matrix derivative–based approaches, and bone grafting—frequently yield variable outcomes despite standardized surgical techniques (117). Sites characterized by residual inflammation or recurrent disease activity are particularly prone to incomplete regeneration or relapse, suggesting that unresolved host-mediated signaling states limit reparative responses (118).

**Figure 4:**
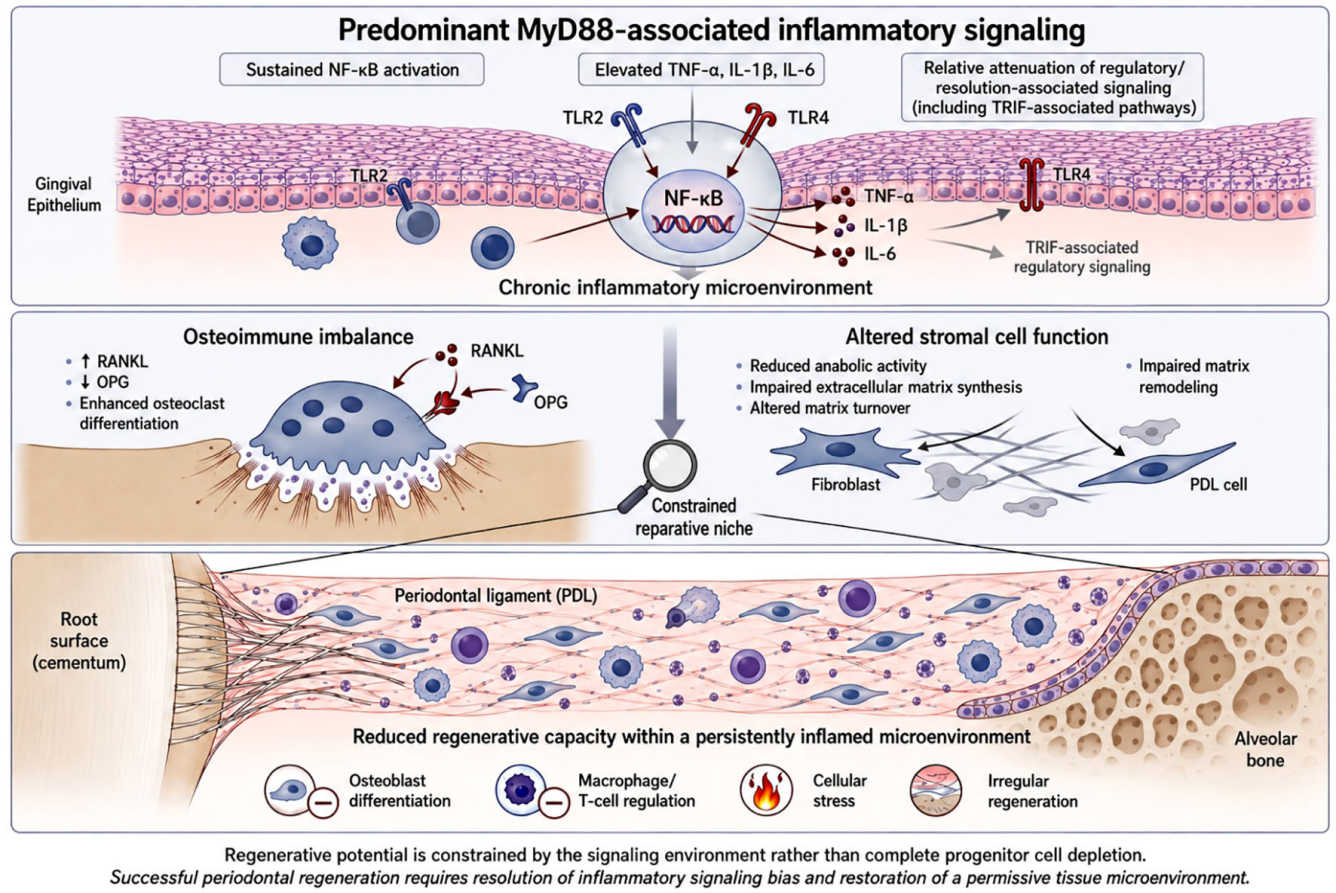
Conceptual model illustrating how persistent TLR-associated inflammatory signaling bias may constrain periodontal regeneration.

From a signaling perspective, regenerative failure in periodontitis can be understood as a downstream consequence of unresolved innate immune activation (2). Dominance of MyD88-dependent TLR signaling sustains inflammatory pathways while potentially limiting engagement of regulatory and resolution-associated programs required for tissue repair, including specialized pro-resolving mediator pathways, resolution-associated macrophage functions, and signaling networks involved in restoration of tissue homeostasis (119). In this context, regenerative interventions applied in the presence of persistent inflammatory bias are unlikely to achieve predictable success, regardless of the intrinsic properties of the applied biomaterials (120) (**Figure 4**).

Sustained predominance of MyD88-associated inflammatory signaling downstream of surface Toll-like receptors, particularly TLR2 and TLR4, is associated with prolonged NF-κB activation, elevated production of pro-inflammatory cytokines, and relative attenuation of regulatory and resolution-associated signaling pathways, including TRIF-associated regulatory signaling. The resulting chronic inflammatory microenvironment promotes osteoimmune imbalance characterized by increased RANKL expression, reduced osteoprotegerin activity, and enhanced osteoclast differentiation. Concurrently, resident stromal cells exhibit altered functional behavior, including reduced anabolic activity, impaired extracellular matrix synthesis, and disrupted matrix remodeling. Together, these changes contribute to the formation of a constrained reparative niche within a persistently inflamed microenvironment. Periodontal stem and progenitor cell function may be limited under these conditions, with impaired osteogenic differentiation, reduced immunomodulatory capacity, and increased cellular stress responses despite continued cellular presence. Collectively, these mechanisms suggest that regenerative potential may be constrained by the signaling environment rather than complete depletion of reparative cell populations.

## 9 oll-like receptors and periodontal stem/progenitor cells

Periodontal stem and progenitor cells are central to tissue homeostasis and regenerative responses following injury (7). These cells reside at the interface between structural integrity and immune surveillance and are therefore continuously exposed to microbial and inflammatory cues (7, 121). Their capacity to sense and integrate such signals is mediated, in part, by expression of functional Toll-like receptors, positioning them as active participants in innate immune regulation rather than passive targets of inflammation (7).

### 9.1 TLR expression profiles in periodontal stem and progenitor cells

Periodontal ligament stem cells express a broad repertoire of TLRs, including surface receptors such as TLR1, TLR2, TLR4, TLR5, and TLR6, as well as endosomal receptors including TLR3 and TLR9 (7). Gingival mesenchymal stem cells display a comparable, though not identical, expression pattern, reflecting their localization within an environment of constant microbial exposure (8, 13). TLR expression in these populations is dynamic and influenced by inflammatory priming, microbial ligands, and cytokine exposure, resulting in context-dependent changes in responsiveness (5, 7, 8, 13).

### 9.2 Effects of TLR activation on survival, proliferation, and migration

TLR engagement exerts profound effects on fundamental stem cell functions (7, 8, 13). Controlled or transient activation of selected pathways can enhance cell survival and promote migration toward sites of tissue injury (8). In particular, activation of endosomal TLR3 has been associated with increased resistance to inflammatory stress and enhanced paracrine activity through TRIF-dependent signaling (8). In contrast, chronic or excessive activation of surface TLRs, especially TLR2 and TLR4 (122), promotes inflammatory stress responses, increases susceptibility to apoptosis or senescence, and limits proliferative and migratory capacity.

### 9.3 TLR signaling and lineage commitment

TLR signaling also influences lineage commitment, a key determinant of regenerative outcome (8). Sustained activation of TLRs such as TLR2 and TLR4 suppresses osteogenic differentiation through inflammatory signaling cascades that interfere with bone-forming programs (67). However, this effect is not uniform; under controlled conditions, TLR engagement can support osteogenic or immunomodulatory phenotypes, depending on ligand characteristics, signal duration, and cellular context as seen with TLR3 (8, 27). This duality highlights that TLR signaling modulates lineage decisions in a highly context-dependent manner (67).

### 9.4 Immunomodulatory functions of TLR-primed stem cells

Beyond differentiation, periodontal stem and progenitor cells exert immunomodulatory effects shaped by TLR engagement (123). Appropriate stimulation, such as seen with TLR3 can induce expression of regulatory mediators such as indoleamine 2,3-dioxygenase, prostaglandin E2, and interleukin-10, enabling suppression of excessive T-cell activation and modulation of macrophage polarization (124). Persistent MyD88-dominant signaling, however, limits these regulatory functions and reinforces inflammatory circuits (125).

### 9.5 Duality of TLR signaling in regeneration

**Figure 5:**
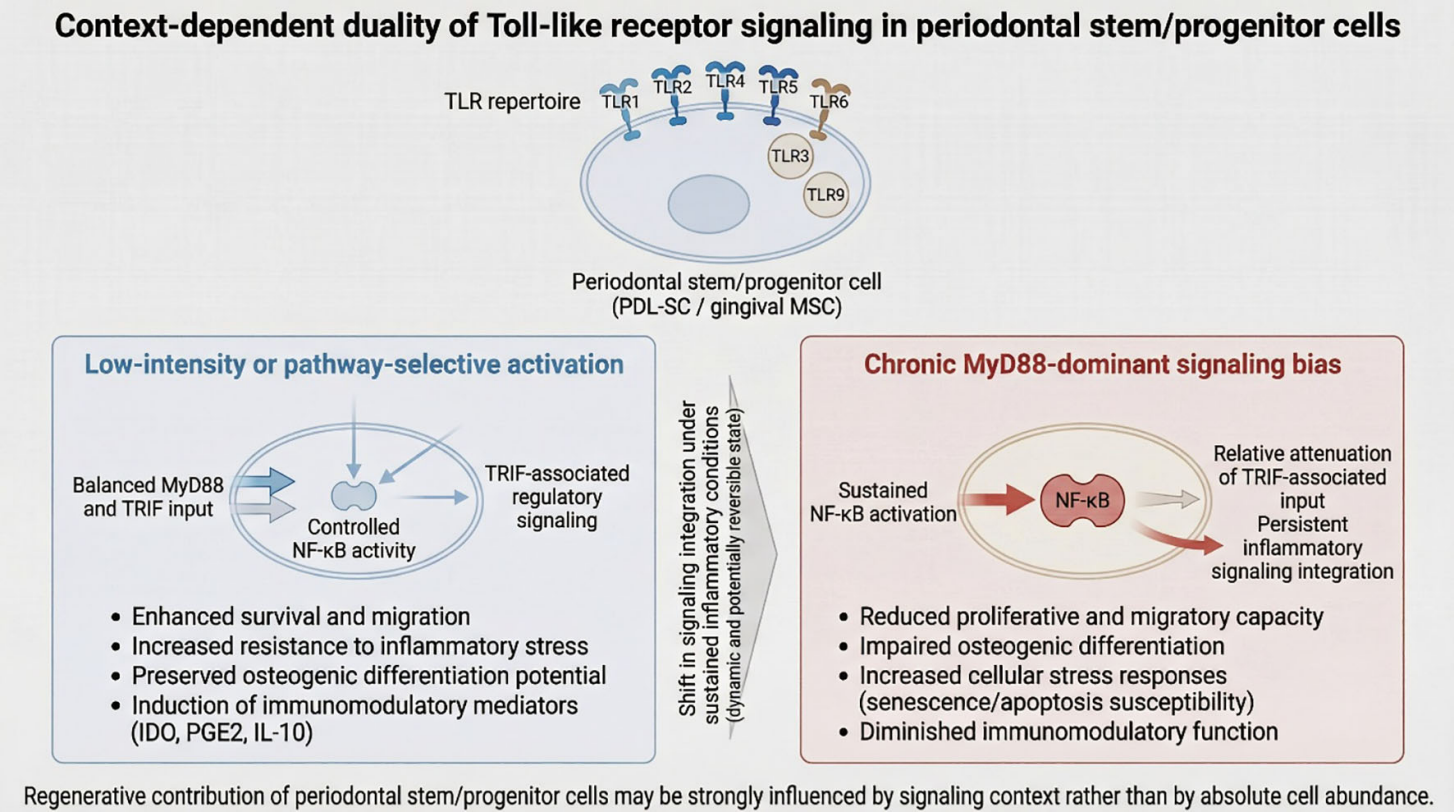
Context-dependent duality of toll-like receptor signaling in periodontal stem/progenitor cells.

Collectively, these findings indicate that TLR signaling in periodontal stem and progenitor cells is inherently dualistic (7). Low-intensity or pathway-selective activation may enhance regenerative competence, whereas chronic inflammatory signaling constrains reparative potential (126). This duality explains why stem cells remain present in chronically inflamed tissues yet fail to support effective regeneration (127) (**Figure 5**).

Periodontal ligament stem cells (PDL-SCs) and gingival mesenchymal stem cells (MSCs) express multiple surface and endosomal Toll-like receptors (TLR1, TLR2, TLR4, TLR5, TLR6, TLR3, and TLR9), enabling direct sensing of microbial and inflammatory signals within the periodontal microenvironment.

Under low-intensity or pathway-selective stimulation, balanced integration of MyD88- and TRIF-associated signaling supports controlled NF-κB activation and preserves regulatory input. In this context, stem/progenitor cells may maintain osteogenic differentiation potential, enhance survival and migration, and induce immunomodulatory mediators such as indoleamine 2,3-dioxygenase (IDO), prostaglandin E2 (PGE2), and interleukin-10 (IL-10).

In contrast, sustained inflammatory conditions promote a MyD88-dominant signaling bias characterized by persistent NF-κB activation and relative attenuation of TRIF-associated pathways. This altered signaling integration is associated with reduced proliferative and migratory capacity, impaired osteogenic differentiation, increased cellular stress responses, and diminished immunomodulatory function.

Collectively, the figure illustrates that the regenerative contribution of periodontal stem/progenitor cells is strongly influenced by the qualitative and temporal context of Toll-like receptor signaling rather than by absolute stem cell abundance.

## 10 herapeutic modulation of Toll-like receptor signaling in periodontitis: opportunities and constraints

The central involvement of Toll-like receptor signaling in periodontal inflammation has stimulated growing interest in host-modulatory strategies aimed at reshaping innate immune pathways (2). In principle, targeting TLR signaling offers the possibility of intervening upstream in the inflammatory cascade rather than focusing exclusively on downstream mediators (128). In practice, however, translation into clinical periodontology remains constrained by several fundamental challenges (10). The periodontium is a barrier tissue exposed to continuous microbial challenge, TLR signaling is essential for antimicrobial defense and wound healing, and innate immune networks exhibit substantial redundancy and adaptability (128). As a result, the therapeutic question is not whether TLR signaling can be altered, but whether it can be selectively modulated to attenuate maladaptive inflammatory persistence while preserving host defense and reparative capacity (128).

Conceptually, two complementary therapeutic directions can be distinguished. The first aims to attenuate chronic pro-inflammatory signaling, particularly MyD88- and NF-κB-dominant outputs downstream of surface TLRs such as TLR2 and TLR4 (14). The second seeks to restore or engage regulatory and reparative signaling arms, including endosomal and TRIF-associated pathways, that may counterbalance inflammatory dominance and support tissue repair (8, 14). These strategies reflect the broader concept advanced in this review: periodontitis is sustained less by TLR activation per se than by a persistent bias in downstream signaling integration (14, 128).

### 10.1 Inhibition of pathological TLR2/TLR4 signaling

Receptor-level antagonism has been explored most extensively for TLR4 (14, 129) and, to a lesser extent, TLR2 (129). Small-molecule inhibitors (130), inhibitory peptides (131), and decoy approaches (132) targeting receptor–adaptor interactions can reduce cytokine production, osteoclastogenic signaling, and alveolar bone loss in preclinical models. These findings provide proof-of-principle that upstream innate immune blockade can influence periodontal tissue outcomes (10). However, broad inhibition of surface TLRs carries inherent risks in a tissue environment that cannot be rendered sterile and relies on intact innate immunity for microbial containment (133).

**Figure 6:**
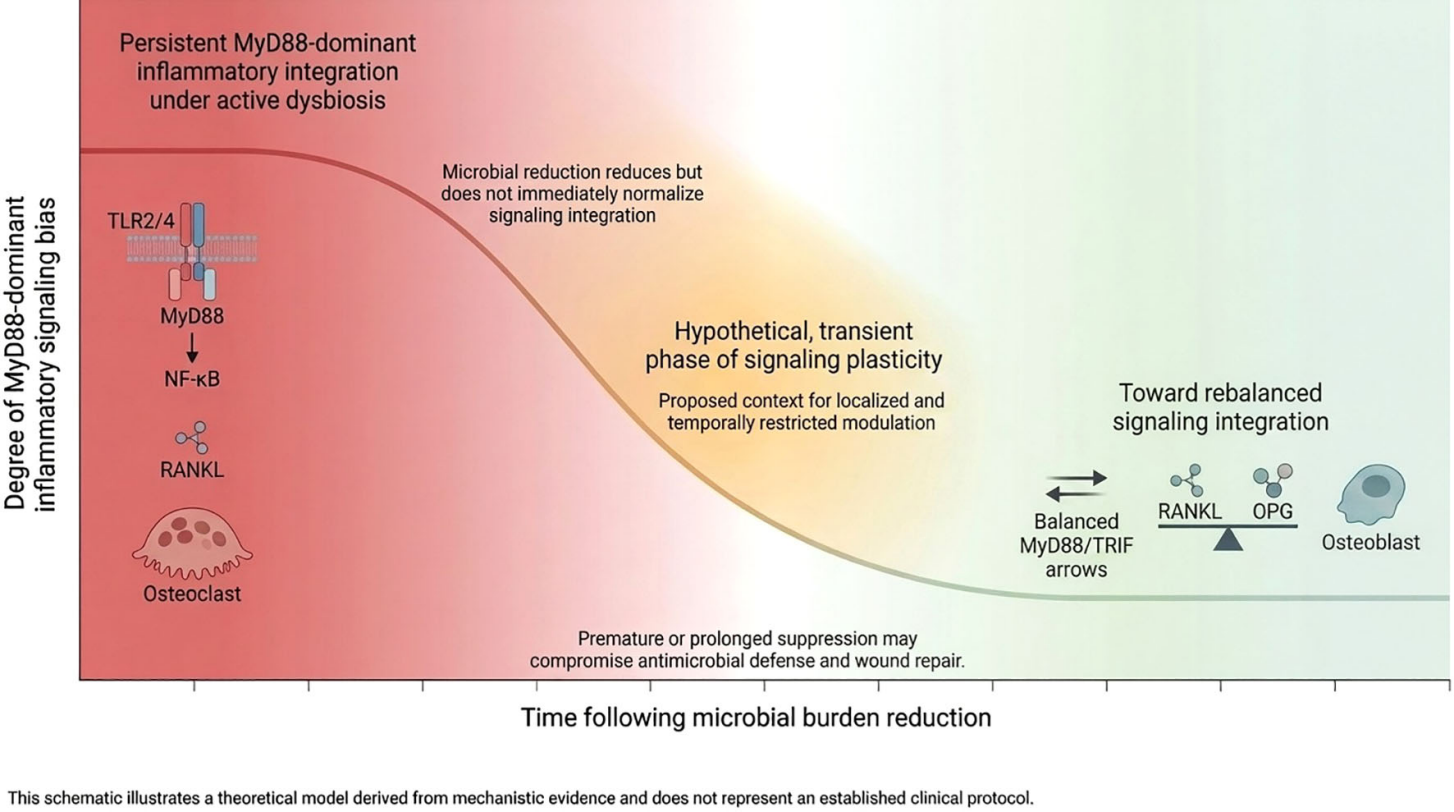
Conceptual model of temporal signaling bias dynamics and a hypothetical modulation window following microbial burden reduction in periodontitis.

Adaptor-level modulation offers a more mechanistically refined approach (14). Because MyD88 integrates signals from multiple TLRs, partial attenuation of MyD88-dependent signaling could, in theory, reduce chronic inflammatory amplification while preserving TRIF-associated programs (14). Nevertheless, MyD88 also plays a critical role in antibacterial defense, and indiscriminate or prolonged inhibition is unlikely to be clinically acceptable (134). Any realistic application would therefore require highly localized, temporally restricted modulation, potentially limited to defined treatment windows such as early post-debridement or post-surgical phases (135) (**Figure 6**).

Persistent periodontitis is characterized by MyD88-dominant Toll-like receptor (TLR) signaling integration under conditions of active dysbiosis, resulting in sustained NF-κB activation, elevated pro-inflammatory mediator production, increased RANKL expression, and osteoclastogenic activity. Mechanical reduction of microbial burden attenuates inflammatory signaling intensity but does not immediately normalize downstream pathway integration, reflecting continued signaling bias within the periodontal microenvironment.

Based on mechanistic observations, a transient phase of signaling plasticity may emerge during early post-reduction healing, during which inflammatory dominance declines but regulatory and reparative pathways are not yet fully restored. This hypothetical transitional context is proposed as a biologically plausible setting for localized and temporally restricted modulation of signaling bias. However, premature or prolonged suppression of innate immune pathways may compromise antimicrobial defense and wound repair.

Progressive restoration toward balanced MyD88- and TRIF-associated signaling integration is associated with normalization of RANKL/OPG dynamics and a regenerative-permissive microenvironment. The schematic represents a theoretical framework derived from mechanistic evidence and does not depict an established clinical protocol.

Targeting downstream signaling pathways, including NF-κB (136) and MAPKs (137), can suppress inflammatory mediator production irrespective of the initiating receptor. While attractive from a pharmacological perspective, this strategy risks nonspecific immunosuppression and may disrupt resolution and repair programs alongside inflammatory pathways (138). Moreover, downstream inhibition does not directly address signaling bias, as suppression of a single pathway does not necessarily restore regulatory or reparative arms of TLR signaling (14).

### 10.2 Restoring regulatory and reparative TLR signaling programs

A conceptually distinct strategy focuses on engaging or restoring TLR-associated pathways that favor immune regulation and tissue repair rather than suppressing signaling globally (27). Endosomal TLR signaling, particularly through TLR3 and TRIF-associated pathways (8, 27), has been implicated in modulating stromal and progenitor cell behavior, stress adaptation, and immunoregulatory function. In periodontal mesenchymal stem and progenitor cells, controlled activation of TLR3 has been shown to influence differentiation trajectories, stemness properties, and immunomodulatory mediator expression under defined conditions (8, 13, 27).

These observations support a “rebalancing” hypothesis in which restoration or transient engagement of regulatory signaling arms may counteract maladaptive MyD88-dominant signaling, particularly within stromal and progenitor compartments (8). However, agonist-based strategies carry substantial risk if applied without precise control (46). Depending on dose, ligand properties, timing, and cellular context, TLR agonists can exacerbate inflammation rather than promote resolution (46). Consequently, plausible translational applications are likely limited to controlled settings such as ex vivo priming of cells prior to implantation (46), incorporation into biomaterials with tightly regulated release kinetics (138), or narrowly timed application following effective microbial load reduction (10).

### 10.3 Localized and biomaterial-guided immune modulation

Given the limitations of systemic immunomodulation (139), localized delivery is central to any realistic therapeutic strategy targeting TLR signaling in periodontitis (140). Biomaterial-based platforms, including hydrogels, nanoparticles, and functionalized scaffolds, offer the ability to confine immune modulation spatially and temporally within periodontal defects (141). Such systems can integrate structural support with controlled presentation of immunomodulatory cues, thereby shaping the local regenerative niche (142).

From a mechanistic perspective, biomaterials may be designed to blunt excessive inflammatory signaling during early healing phases or to support regulatory and reparative programs during later stages of regeneration (143). In the context of stem and progenitor cell–based approaches, constructs incorporating cells preconditioned under defined TLR cues may enhance resilience to inflammatory stress and promote immunoregulatory activity (104). These strategies are consistent with the concept that regenerative failure reflects an unfavorable signaling environment rather than absence of regenerative cell populations (115).

### 10.4 Gene- and RNA-based approaches to modulating TLR networks

Gene- and RNA-based strategies offer conceptual precision by targeting specific signaling nodes or regulatory checkpoints within TLR pathways (144, 145). Approaches such as siRNA-mediated knockdown of pro-inflammatory adaptors (146), CRISPR-based editing (147), or modulation of microRNAs involved in negative feedback regulation (148) represent powerful experimental tools. However, effective and safe delivery within periodontal tissues remains a major obstacle (149). At present, ex vivo manipulation of cells prior to implantation appears more feasible than direct *in vivo* genome or transcriptome editing (149).

### 10.5 Targeted protein degradation and next-generation modality toolkits: PROTACs, PROTABs, and beyond

Conventional inhibition of TLRs or TLR signaling components typically blocks enzymatic activity while leaving non-catalytic scaffolding functions intact (14, 150). This limitation is particularly relevant for adaptor proteins and signaling hubs within TLR pathways (14, 150). Targeted protein degradation approaches offer a distinct mechanistic strategy by eliminating the protein itself, thereby disrupting both catalytic and structural roles (150). In the context of periodontal research, such approaches are best viewed as experimental tools for establishing causality within complex innate immune networks rather than as immediate therapeutic solutions (14, 150).

Proteolysis-targeting chimeras (PROTACs) are bifunctional molecules that recruit target proteins to E3 ubiquitin ligases, leading to ubiquitination and proteasomal degradation (150, 151). Applied to TLR signaling, PROTACs enable direct testing of whether sustained inflammatory states depend on continuous presence of specific signaling nodes such as MyD88, IRAK4, or TRAF6 (14, 19, 150). By allowing time-resolved removal of these proteins, degradation strategies can distinguish between signaling events required for initiation of inflammation and those necessary for its maintenance (150, 151). For pathways characterized by redundancy and feedback regulation, degradation offers advantages over classical inhibition (152). Removal of a signaling scaffold can collapse entire signalosomes and reveal dependencies that are masked when enzymatic activity alone is inhibited (153). This capability is particularly relevant for interrogating signaling bias, as it allows assessment of whether biased inflammatory states are actively maintained by specific hubs or become self-sustaining once established (154–156).

Despite these conceptual strengths, application of PROTACs in periodontal research faces substantial limitations. Most available degraders have been developed for oncology or systemic immunology (157) and have not been optimized for primary gingival stromal cells periodontal ligament cells, or organotypic periodontal models. Cell permeability, stability, off-target proteotoxic stress, and variability in E3 ligase expression represent significant challenges (158). Moreover, effective delivery within periodontal tissues remains unresolved, precluding systemic or chronic application. Antibody-guided degradation strategies, including PROTAB-like concepts, offer the theoretical advantage of cell-type specificity by coupling target recognition to surface markers (159). In heterogeneous periodontal tissues, such specificity would be desirable (160). However, barriers related to tissue access, protease activity, and local delivery currently limit feasibility (161). As with PROTACs, the primary value of these approaches lies in mechanistic interrogation rather than near-term clinical translation (162).

Importantly, targeted degradation technologies contribute to periodontal research even in the absence of direct therapeutic application. They enable time-resolved causal analysis, facilitate dissection of cell-type–specific signaling dependencies, reveal compensatory network responses, and support identification of candidate biomarkers associated with signaling bias (163, 164). Insights gained through such approaches may inform development of more clinically feasible strategies, including localized inhibitors, RNA-based modulation, or biomaterial-guided delivery systems (163, 164).

Taken together, targeted protein degradation and related next-generation modalities expand the experimental toolkit available to study TLR signaling bias in periodontitis. Their current role is to enhance mechanistic resolution and support hypothesis-driven research aimed at identifying tractable signaling nodes. Whether these insights ultimately translate into clinical interventions will depend on advances in biomarker discovery, delivery technologies, and precise temporal control of immune modulation.

### 10.6 Clinical relevance and translational outlook

At present, TLR-focused interventions have limited direct applicability in routine periodontal therapy (10, 165). Their immediate clinical value lies in explaining variable treatment responses, motivating biomarker discovery for inflammatory signaling states, and informing rational design of localized adjunctive therapies (10, 165). A realistic translational roadmap will require identification of measurable markers of signaling bias, demonstration that local modulation can reduce maladaptive inflammation without compromising host defense, and development of delivery platforms compatible with periodontal practice (166). This plan will require the application of insights from experimental approaches to protein degradation to human tissue models to validate signaling nodes before clinical translation.

## 11 Natural compounds as context-dependent modulators of toll-like receptor signaling in periodontitis

In addition to synthetic inhibitors and molecularly targeted strategies, increasing experimental evidence indicates that selected natural compounds can modulate Toll-like receptor (TLR) signaling in a context-dependent manner (167). Importantly, most naturally derived agents do not function as classical receptor antagonists and do not abrogate innate immune signaling. Instead, they exert partial, pleiotropic effects on TLR expression, responsiveness, or downstream signal integration, leading to attenuation of excessive inflammatory outputs while preserving essential host-defense functions (167, 168).

Experimental studies across innate immune systems demonstrate that a variety of plant-derived and bioactive compounds influence TLR-associated pathways, most commonly TLR4-dependent inflammatory signaling (167, 169, 170). However, although multiple natural compounds have been reported to modulate Toll-like receptor signaling in experimental systems, direct evidence in periodontal tissues remains limited, with the notable exception of thymoquinone-mediated regulation of TLR expression in human gingival mesenchymal stem/progenitor cells (5). This finding provides a rare example of direct TLR modulation within a periodontal-relevant cellular context and underscores the tissue specificity of innate immune signaling responses (5).

Beyond periodontal cell systems, multiple natural compounds have been reported to attenuate TLR-driven inflammatory signaling in immune and non-oral tissues, primarily through modulation of TLR4-associated pathways (167, 169, 170). Reported mechanisms include reduced recruitment of MyD88-dependent signaling components (171), attenuation of NF-κB and MAPK activation (172), and indirect regulation of inflammatory signaling via redox-sensitive processes (167). Importantly, these effects are highly dependent on concentration, exposure duration, and cellular context, underscoring their pleiotropic and non-selective nature (167).

A defining characteristic of natural compound–mediated TLR modulation is its context sensitivity (167). Depending on experimental conditions, the same compound may exert anti-inflammatory, immunomodulatory, or stress-adaptive effects (5, 173). This property aligns with the emerging concept that periodontal inflammation is sustained by maladaptive signaling integration rather than by uniform receptor overactivation (174). Accordingly, natural compounds are best conceptualized as low-intensity modulators capable of reshaping signaling bias within innate immune networks rather than as precise inhibitors of discrete molecular targets (173).

Despite promising mechanistic observations, important limitations constrain the translational interpretation of these findings. Most studies have been conducted in simplified *in vitro* systems or non-periodontal animal models, and pharmacokinetic behavior within periodontal tissues remains poorly defined. Moreover, the molecular targets of many natural compounds are incompletely characterized, limiting reproducibility and mechanistic resolution (175). Consequently, current evidence does not support the use of natural compounds as stand-alone therapeutic interventions for periodontitis (173, 176).

Within the conceptual framework of this review, the primary value of natural compounds lies in their contribution to mechanistic understanding. By demonstrating that TLR-associated inflammatory outputs can be modulated without abolishing innate immune responsiveness, these studies reinforce the notion that periodontal inflammation may be amenable to rebalancing rather than global immune suppression (5). Insights gained from such modulation may inform the development of more selective, controllable host-modulatory strategies, particularly when integrated with localized delivery systems or regenerative approaches.

In this respect, the main importance of research on natural compounds lies not in the development of a therapeutic approach but in providing proof-of-principle that the biased signaling state of the inflamed periodontium is pharmacologically accessible and can be shifted towards a more regulated state.

Collectively, the diverse experimental and conceptual strategies for modulating Toll-like receptor signaling in periodontitis—ranging from receptor antagonism to regulatory engagement, biomaterial-guided delivery, RNA-based approaches, targeted protein degradation, and natural compound–mediated modulation—are summarized in **Table 3**.

**Table 3:** Strategies for therapeutic modulation of TLR signaling in periodontitis, including targets, mechanisms, advantages, and limitations.

Strategy category	Primary target(s)	Intended mechanism	Potential advantages	Key limitations
TLR2/TLR4 antagonists	TLR2, TLR4	Block receptor–ligand interaction or adaptor recruitment	Direct reduction of inflammatory cytokine output; proof-of-concept efficacy in preclinical models	Risk of impaired antimicrobial defense; lack of selectivity; unsuitable for chronic use
Adaptor-level modulation	MyD88, IRAK4, TRAF6	Partial attenuation of signal integration	Mechanistically refined targeting of inflammatory amplification	Central role in host defense; pathway redundancy; delivery challenges
Downstream pathway inhibition	NF-κB, MAPKs	Suppression of inflammatory transcriptional programs	Broad anti-inflammatory effect	Nonspecific immunosuppression; disruption of resolution and repair pathways
Regulatory pathway activation	TLR3/TRIF-associated signaling	Engagement of immunoregulatory and stress-adaptive programs	Potential to rebalance signaling without global inhibition	Strong context dependence; risk of inflammatory exacerbation
Biomaterial-guided modulation	Local immune niche	Spatial and temporal control of immune cues	Localized action; integration with regeneration	Complexity of design; limited clinical validation
RNA-based approaches	TLRs, adaptors, negative regulators	Downregulation or feedback restoration	High specificity in experimental systems	Delivery, stability, off-target effects
Targeted protein degradation	TLRs, MyD88, IRAK4, TRAF6	Removal of signaling hubs	High mechanistic resolution; causality testing	Experimental only; delivery and toxicity constraints
Natural compound–mediated modulation	TLR expression/MyD88–NF-κB axis (context-dependent)	Partial attenuation or rebalancing of signaling bias	Pleiotropic modulation; potential preservation of host defense	Low specificity; variable bioavailability; limited periodontal evidence Lack of mechanistic clarity and reproducible standardization

## 12 TLR systemic integration and cross-talk in periodontitis and systemic diseases

The conceptual framework established throughout this review, positioning periodontitis as a disease of imbalanced TLR signaling integration, assumes particular relevance when considering the well-documented epidemiological links between periodontitis and systemic inflammatory diseases. Cardiovascular disease, type 2 diabetes mellitus, rheumatoid arthritis, and neurodegenerative diseases have been firmly associated with periodontitis in large-scale observational studies. Although these associations are complex in nature, there is a growing body of evidence that Toll-like receptor signaling plays a pivotal role in mediating the link between local periodontitis and systemic inflammation (2). These epidemiological associations are increasingly supported by mechanistic evidence implicating Toll-like receptor signaling as a shared immunobiological axis. Across cardiovascular (177), Metabolie (178), autoimmune (4), neurodegenerative (179), and renal disorders (180), periodontal pathogens and their ligands engage distinct TLR subsets in extraoral tissues, leading to context-dependent activation of downstream inflammatory pathways. Importantly, the biological impact of circulating TLR ligands depends not only on receptor presence but also on cell-type–specific signal integration (181), local microenvironmental cues (182), and host genetic susceptibility (183). The principal receptor pathways, downstream mediators, implicated periodontal pathogens, and translational implications across major systemic conditions are summarized in **Table 4**.

**Table 4:** TLR-mediated systemic cross-talk in periodontitis: disease-specific mechanisms, key mediators, and clinical implications.

Systemic disease	Key TLRs involved	Molecular mechanism	Key downstream mediators	Periodontal bacteria implicated	Clinical implication
Cardiovascular Disease (CVD)	TLR2, TLR4	TLR4 activation on endothelial cells by periodontal LPS → NF-κB signaling → upregulation of VCAM-1, ICAM-1; macrophage TLR activation → foam cell formation → atherogenesis; nitric oxide imbalance → endothelial dysfunction	IL-1β, IL-6, TNF-α, CRP; adhesion molecules (VCAM-1, ICAM-1); oxidative stress markers	P. gingivalis, F. nucleatum, T. denticola	Periodontal treatment may reduce cardiovascular risk; TLR4 as a biomarker of systemic inflammatory burden
Type 2 Diabetes Mellitus (T2DM)	TLR2, TLR4, TLR5	TLR4-MyD88/TRIF pathway → gut microbiota dysbiosis → systemic insulin resistance; Th17/Treg imbalance + STAT3/SOCS3 → impaired insulin signaling; TLR2/TLR4 on adipose macrophages → pro-inflammatory polarization; RAGE-ROS/NF-κB → β-cell dysfunction	TNF-α, IL-1β, IL-6; AGEs; SOCS3; impaired insulin receptor substrate phosphorylation	P. gingivalis (LPS, flagellin); F. nucleatum; periodontal dysbiosis-associated gut pathogens	Bidirectional relationship; periodontal therapy may improve glycemic control; TLR4 and gut microbiome as intervention targets
Rheumatoid Arthritis (RA)	TLR2, TLR4, TLR9	PPAD-mediated protein citrullination → generation of ACPA neo-antigens; PAD4 from neutrophils/NETs; TLR4 activation → host autoantigen susceptibility; TLR9 activation by circulating bacterial DNA → enhanced autoimmune response; periodontal tissue as dual source of autoantigens + TLR adjuvants	ACPA; RF; citrullinated proteins; IL-17, IL-6, TNF-α; RANKL → osteoclastic bone erosion	P. gingivalis (PPAD enzyme, LPS); oral bacterial DNA	Shared immunopathology; ACPA screening may identify at-risk patients; periodontal therapy as adjunct in RA management
Neurodegenerative Disease (Alzheimer’s Disease)	TLR2, TLR4	Hematogenous/trigeminal/oral-gut routes → CNS entry; gingipains + LPS → neuroinflammation + Aβ aggregation + tau hyperphosphorylation; microglial TLR2/TLR4 activation → pro-inflammatory phenotype → neuronal damage; oral-gut-brain axis via gut microbiome modulation	IL-1β, IL-6, TNF-α; amyloid-β (Aβ); tau; NF-κB; NLRP3 inflammasome; impaired microglial Aβ clearance	P. gingivalis (gingipains, LPS); oral-gut pathogens via saliva ingestion	Emerging oral-brain axis; early periodontal intervention may attenuate neuroinflammatory burden; gingipain inhibitors under investigation
Chronic Kidney Disease (CKD)	TLR4 (primary)	TLR4 on glomerular mesangial cells, endothelial cells, renal tubular epithelium → MyD88-NF-κB pathway → renal inflammation; NF-κB/NLRP3-ferroptosis axis in mesangial cells; uremic DAMPs (hyaluronan, heparan sulfate, AGEs) → self-reinforcing TLR4 activation; TLR4 rs2149356 polymorphism → genetic susceptibility	IL-1β, IL-6, TNF-α; KIM-1; creatinine; NF-κB; NLRP3; uremic DAMPs	P. gingivalis (LPS, virulence factors)	Periodontal therapy reduces renal TLR4/NF-κB expression; TLR4 rs2149356 as genetic risk marker; plaque control as modifiable risk factor for CKD progression

ACPA, anti-citrullinated protein antibodies; AGEs, advanced glycation end products; Aβ, amyloid-beta; CKD, chronic kidney disease; CNS, central nervous system; CVD, cardiovascular disease; DAMPs, damage-associated molecular patterns; ICAM-1, intercellular adhesion molecule-1; KIM-1, kidney injury molecule-1; LPS, lipopolysaccharide; NETs, neutrophil extracellular traps; NF-κB, nuclear factor kappa B; NLRP3, NOD-like receptor protein 3; PAD4, peptidylarginine deiminase 4; PPAD, Porphyromonas gingivalis peptidylarginine deiminase; RA, rheumatoid arthritis; RAGE, receptor for advanced glycation end products; RANKL, receptor activator of nuclear factor kappa-B ligand; RF, rheumatoid factor; SOCS3, suppressor of cytokine signaling 3; STAT3, signal transducer and activator of transcription 3; T2DM, type 2 diabetes mellitus; TLR, Toll-like receptor; TNF-α, tumor necrosis factor-alpha; VCAM-1, vascular cell adhesion molecule-1.

### 12.1 Mechanisms of systemic dissemination

Periodontal tissues, especially those that are inflamed, offer numerous sites through which bacterial products, activated immune cells, and soluble inflammatory mediators can gain access to the systemic circulation (184). The ulcerated pocket epithelium, a hallmark of periodontitis, represents a compromised barrier through which bacteria and bacterial products can pass directly into the underlying vasculature (185). Common oral functions, such as chewing and brushing teeth, cause transient bacteremias of periodontal pathogens, such as Porphyromonas gingivalis, Fusobacterium nucleatum, and other species associated with periodontal dysbiosis (186).

Most importantly, TLR ligands themselves, including lipopolysaccharide (LPS), bacterial lipoproteins, and microbial nucleic acids, may gain access to the systemic circulation in patients with periodontitis (187, 188). Once present in the circulation, microbial products and other inflammatory mediators can interact with TLRs expressed on endothelial cells (188), hepatocytes (189), adipocytes (190), and immune cells (191), thereby initiating inflammatory signaling cascades at sites distant from the periodontal lesion (187, 188).

### 12.2 Cardiovascular disease: TLR-mediated vascular inflammation

One of the most well-investigated relationships between periodontal disease and systemic conditions is that of periodontitis and cardiovascular disease. Periodontal bacteria induce endothelial oxidative stress and systemic inflammation through Toll-like receptor activation, NF-κB signaling, and nitric oxide imbalance, leading to endothelial dysfunction and atherogenesis (8).

Activation of TLR4 on endothelial cells by periodontal LPS leads to the expression of adhesion molecules such as vascular cell adhesion molecule-1 and intercellular adhesion molecule-1, facilitating the entry of monocytes into the atherosclerotic lesion. Simultaneously, TLR activation on macrophages in the plaque enhances foam cell formation through increased uptake of lipoproteins and reduced cholesterol efflux, hastening the accumulation of lipid-laden macrophages, which are typical of early atherosclerotic lesions (8). The low-grade inflammatory environment perpetuated by periodontal TLR activation thus plays a role in the pathogenesis of atherosclerosis through several overlapping pathways.

### 12.3 Metabolic disorders: TLRs and insulin resistance

The relationship between type 2 diabetes mellitus and periodontitis has been clearly demonstrated to be bidirectional, and TLR signaling plays an important role in this connection. Several TLR-mediated mechanisms have been identified to link periodontal infection with metabolic disorders. The periodontitis-induced dysbiosis of gut microbiota activates the TLR4-MyD88/TRIF signaling pathway, which plays a role in the systemic insulin resistance caused by periodontitis (3, 9). This gut-associated pathway provides another mechanism by which periodontitis-induced inflammation causes metabolic disorders.

In addition, periodontal pathogen LPS overcomes the balance between T helper 17 (Th17) and regulatory T (Treg) cells, and the subsequent activation of the STAT3/SOCS3 pathway suppresses insulin signaling (9). Flagellin from periodontal pathogens has been shown to be involved in the inhibition of insulin secretion (192), while advanced glycation end products induce RAGE-ROS/NF-κB pathway activation, causing dysfunction of pancreatic β-cells (193). Adipose tissue macrophages, which accumulate in obesity and cause metabolic inflammation, are sensitive to periodontal TLR ligands. Activation of TLR2 and TLR4 on these cells promotes their polarization into pro-inflammatory cells, which increase the production of cytokines such as TNF-α and IL-1β, causing insulin signaling dysfunction in neighboring adipocytes and hepatocytes (194, 195).

### 12.4 Rheumatoid arthritis: convergent TLR pathways and autoimmunity

The link between periodontitis and Rheumatoid Arthritis (RA) has recently attracted considerable interest, with P. gingivalis being proposed as a putative underlying mechanism due to its distinctive ability to citrullinate proteins through the enzyme peptidylarginine deiminase (PPAD) (11). In addition to this particular mechanism, TLR signaling is involved in the periodontitis-RA association through a variety of convergent pathways.

Periodontal tissues are a source of both citrullinated antigens and TLR ligands of relevance to RA pathogenesis (4). Citrullination is mediated by two different enzyme sources: peptidylarginine deiminase 4 (PAD4) from periodontal neutrophils and neutrophil extracellular traps, and the particular PPAD enzyme of P. gingivalis. While PAD4-citrullinated host peptides are physiological, PPAD-citrullinated peptides are generated under pathological conditions as neo-antigens (11). Periodontitis-induced endotoxemia may enhance host susceptibility to autoantigens through TLR4 activation, contributing to the initiation and perpetuation of RA. Moreover, circulating periodontal bacterial DNA is phagocytosed by immune cells, triggering TLR9 activation and enhancing susceptibility to autoantigens (4). Periodontal tissues are thus a source of both autoantigens (citrullinated proteins) and adjuvants (TLR ligands) necessary for the induction and maintenance of autoimmune responses.

### 12.5 Neurodegenerative diseases: TLRs and the oral-brain axis

There is emerging evidence that periodontal pathogens, especially P. gingivalis, are involved in the pathogenesis of neurodegenerative diseases, including Alzheimer’s disease (5, 11). P. gingivalis and its virulence factors, such as gingipains, have been found in the brains of Alzheimer’s disease patients, and animal studies have shown that oral infection with P. gingivalis results in colonization of the brain, neuroinflammation, and amyloid deposition.

Periodontal pathogens enter the brain via hematogenous, trigeminal, or oral-intestinal routes, causing neuroinflammation, β-amyloid (Aβ) aggregation, and tau hyperphosphorylation due to the action of virulence factors such as gingipains and LPS (5). TLR signaling plays a role in this oral-brain axis by several mechanisms. Circulating TLR ligands of periodontal origin can diffuse into the brain or stimulate endothelial cell TLRs, triggering inflammatory responses in the central nervous system. Microglia, the brain-resident macrophages, express TLR2 and TLR4, and respond to periodontal pathogens and their components by undergoing pro-inflammatory phenotypic conversions that contribute to neuronal damage and amyloid clearance dysfunction (11). The chronic, low-level activation of TLRs in the brain, due to the presence of circulating periodontal ligands or direct bacterial access, may play a role in the chronic neuroinflammation observed in neurodegenerative diseases.

A new paradigm is the “oral-gut-brain axis,” in which periodontal pathogens ingested in saliva migrate to the gut, where they alter the gut microbiome and, in turn, affect the central nervous system via gut-brain axis signaling. This oral-gut-brain axis is a new mechanism by which periodontal TLR signaling could have distant effects (5).

### 12.6 Chronic kidney disease: TLR-mediated renal inflammation and genetic susceptibility

The link between periodontitis and chronic kidney disease (CKD) is one of the most important manifestations of the relationship between oral and systemic health, with inflammation, especially TLR-mediated inflammation, playing the key role as the ‘mechanistic link’ (196). Periodontal bacteria, especially Porphyromonas gingivalis, and their virulence factors are released into the circulation through ulcerated pocket epithelium, resulting in the activation of TLR4 on glomerular mesangial cells, endothelial cells, and renal tubular epithelium (197). This activation triggers the MyD88-dependent NF-κB pathway, culminating in the production of pro-inflammatory cytokines (IL-1β, IL-6, TNF-α), adhesion molecules, and the infiltration of inflammatory cells into the kidney (197).

Experimental models using animals have offered direct causal proof: the oral route of P. gingivalis administration causes CKD in mice due to interactions between the NF-κB/NLRP3 pathway and ferroptosis in glomerular mesangial cells (198), while ligature-induced periodontitis in obese rats leads to a marked augmentation of renal TLR4 and NF-κB mRNA levels, accompanied by tubular injury and structural damage, vacuolar degeneration, and hypercreatinemia and kidney injury molecule-1 (KIM-1) levels (197). Notably, these changes are reversible with periodontal treatment—plaque control reduces renal TLR4/NF-κB expression and improves kidney injury, thus establishing causality (197). In addition to the direct inflammatory process, genetic predisposition plays a role in modulating this association. The TLR4 rs2149356 polymorphism has been linked to both periodontitis and end-stage renal disease (ESRD) in Egyptian populations, with the TT genotype being associated with increased inflammatory severity and suggesting gene-environment interactions that may confer susceptibility to more aggressive disease (92).

The activation of TLR4 in the kidney is further enhanced by the presence of endogenous ligands such as hyaluronan, heparan sulfate, and advanced glycosylation end products, which are present in the uremic state and continue to drive the inflammatory response even when microbial loads are decreased (92). This self-reinforcing cycle, in which periodontal TLR4 activation drives renal inflammation and the uremic environment enhances TLR4 responsiveness, is consistent with the bidirectional and self-reinforcing nature of periodontal-renal axis disease. Taken together, these data place TLR4 signaling at the hub of periodontitis and CKD progression and suggest periodontal therapy as a potential modifiable risk factor for preserving renal function (196, 199).

### 12.7 Shared mechanisms and clinical implications

Across these systemic diseases, several common mechanistic themes emerge. First, bacterial translocation may allow periodontal pathogens or their components to access distant tissues and stimulate local TLRs (4). Second, systemic inflammation may be amplified by circulating TLR ligands of periodontal origin, which can activate TLRs on immune and parenchymal cells throughout the body (4). Third, migration of immune cells previously exposed to periodontal inflammatory signals may contribute to dissemination of TLR-associated immune responses beyond the oral environment (200). Fourth, inflammatory mediators produced in response to periodontal TLR signaling may exert endocrine-like effects on distant target organs (201).

Notably, the model of biased signaling described throughout this review may also be relevant to systemic disease conditions. The biological consequences of TLR stimulation at distant sites are not determined solely by ligand availability but also by cell-type–specific signal integration, the intensity and duration of exposure, local tissue microenvironments, and host genetic susceptibility factors. This perspective helps explain why only a subset of patients with periodontitis develop specific systemic complications despite similar microbial challenges. It also suggests that modulation of maladaptive TLR signaling pathways may represent a potential strategy for reducing the systemic consequences associated with periodontal inflammation (2).

## 13 Conclusions

Toll-like receptors occupy a central position in the pathobiology of periodontitis by linking microbial recognition to host inflammatory and immunoregulatory responses. The evidence synthesized in this review indicates that TLRs do not function as uniformly destructive drivers of periodontal tissue breakdown. Instead, their biological impact is determined by signaling context, downstream pathway integration, and cell-type–specific responses within the periodontal microenvironment.

Chronic periodontitis may be viewed as a condition in which maladaptive bias in innate immune signaling represents a major driver of disease persistence, characterized by persistent predominance of pro-inflammatory, MyD88-dependent pathways and insufficient engagement of regulatory and resolution-associated programs. This imbalance promotes inflammatory persistence, osteoimmune dysregulation, and impaired reparative capacity, even in the presence of effective microbial control.

Importantly, this framework suggests that regenerative failure in periodontitis is driven, at least in part, by immunoregulatory dysfunction rather than solely by a deficiency of regenerative cell populations. Periodontal stem and progenitor cells remain present in chronically inflamed tissues but are functionally constrained by an unfavorable signaling milieu that suppresses their osteogenic and immunomodulatory potential. Restoration of signaling balance is likely to represent an important prerequisite for predictable regeneration.

From a translational perspective, the clinical value of TLR biology currently lies in explaining disease heterogeneity, variable treatment response, and limited regenerative predictability. Direct therapeutic targeting of TLR pathways remains constrained by biological complexity and safety considerations. Future progress will depend on identifying measurable markers of signaling bias, defining critical signaling nodes that sustain inflammatory persistence, and developing localized, temporally controlled strategies that preserve essential host-defense functions.

Emerging experimental approaches, including targeted protein degradation, RNA-based modulation, advanced biomaterial-guided immune regulation, and next-generation molecular toolkits, provide powerful means to establish causality within complex signaling networks. These technologies may facilitate identification of critical signaling nodes, clarify the contribution of less extensively studied receptors such as TLR3, TLR5, TLR7, and TLR9, and improve understanding of how inflammatory and resolution-associated pathways interact within distinct periodontal cell populations. Although not yet suited for routine clinical application, such approaches offer important opportunities to refine mechanistic models and accelerate development of biologically informed host-modulatory strategies.

In conclusion, periodontitis can be viewed as a disorder of dysregulated innate immune signaling in which microbial challenge, host-response heterogeneity, defective resolution, and regenerative impairment converge within a common biological framework. Rather than functioning as uniformly pathogenic receptors, TLRs operate within highly context-dependent signaling networks whose effects vary according to receptor subtype, cellular compartment, and microenvironmental conditions. Future research should focus on defining measurable biomarkers of signaling bias, clarifying cell-type–specific regulatory mechanisms, integrating emerging knowledge regarding non-TLR2/4 family members, and identifying therapeutic windows in which inflammatory responses can be rebalanced without compromising host defense. Such advances will be essential for translating mechanistic insight into precision host-modulatory approaches capable of improving both disease control and regenerative outcomes.
